# A short‐term longitudinal study linking adolescents' metacognition, learning, and social friendship networks

**DOI:** 10.1111/jora.70072

**Published:** 2025-08-28

**Authors:** Mariëtte van Loon, Lydia Laninga‐Wijnen

**Affiliations:** ^1^ Department of Psychology University of Zurich Zurich Switzerland; ^2^ INVEST Flagship University of Turku Turku Finland

**Keywords:** adolescence, control, metacognition, monitoring judgments, peer influence, self‐regulated learning, social networks

## Abstract

Adolescents' metacognitive skills and social relationships play key roles in learning but are often studied in isolation. This study investigated the links between metacognition, learning performance, and classroom friendship networks in a sample of 136 seventh‐grade students from Switzerland (53.8% female; mean age 13.8 years) assessed at two time points 3 months apart. Metacognition was measured on‐task. Monitoring was assessed through confidence judgments, and control through decisions about what to restudy and which responses to submit or withdraw from grading. Participants learned the meanings of Japanese characters (“Kanji”), self‐tested memorization, monitored their performance, made restudy decisions, and submitted selected responses. Social friendship networks were measured with friendship nominations within classrooms. Results showed that decision accuracy strongly predicted Kanji task scores at both time points. Monitoring‐based restudy became a significant predictor of task scores at the second measurement, indicating that participants who strategically restudied items for which confidence was initially low achieved higher scores. No evidence was found for friends influencing adolescents' metacognitive skills. Friends did, however, become more similar in task performance over time, suggesting that peer influence may shape learning processes other than the investigated metacognitive processes. These findings highlight the importance of metacognitive control and friendship dynamics for adolescents' learning outcomes.

## INTRODUCTION

The transition to secondary schools comes together with increasing academic and social challenges. Academically, youth are expected to take greater responsibility in learning, requiring more advanced metacognitive skills– the ability to monitor and control their learning processes (Schneider & Löffler, 2016). Socially, youth are often confronted with an influx of new, unfamiliar peers after the transition, and –ushered in with pubertal changes –they attach more importance to belonging to these peers (e.g., forming friendships; Laninga‐Wijnen & Veenstra, 2021). To date, the role of metacognition and social relationships (friendships) in learning and performance has largely been examined in isolation, and both areas face methodological limitations. For metacognition, task‐based measures which capture behavioral processes during learning are more strongly linked to academic performance than self‐report tools like surveys or questionnaires (Muncer et al., 2022; Ohtani & Hisasaka, 2018). Yet, among adolescents, most research still relies on self‐report instruments (Gascoine et al., 2017). For social relationships, research applying social network analyses (a methodology suitable for testing complex interdependencies) often examined friendship influence on broad academic outcomes like GPA or self‐reported grades (e.g., Wang et al., 2018), potentially overlooking the role of peer influence on real‐time, task‐specific learning processes. In the current study, we combine task‐based process measures of metacognition and learning with social network data. We aim to investigate the role of metacognitive skills in adolescent learning and to better understand how adolescents' friends may shape their metacognitive processes and learning performance.

## EFFECTS OF METACOGNITION ON LEARNING

Models of metacognition emphasize that self‐monitoring of cognitive processes and the resulting control decisions (i.e., actions taken during learning) directly influence learning outcomes (Nelson, 1990). Metacognition is usually divided into declarative metacognition (i.e., awareness of learning strategies) and procedural metacognition (i.e., the ability to monitor and control learning during a task; Schneider & Löffler, 2016). Declarative metacognition is often measured via surveys, while procedural metacognition is assessed through on‐task measures capturing real‐time monitoring and control. Meta‐analyses show on‐task measures relate more strongly to academic achievement than self‐reports, as they better reflect actual learning (Muncer et al., 2022; Ohtani & Hisasaka, 2018). However, a review by Gascoine et al. (2017) shows that research using on‐task measures to investigate adolescent metacognition (ages 12–16) remains limited. Most studies in this age range rely on self‐report tools like surveys and questionnaires, while task‐based approaches account for <10%. This highlights a gap in capturing real‐time metacognitive processes during learning. In particular, studies assessing both monitoring and control remain scarce, as most research on procedural metacognition focuses only on the assessment of monitoring (Baars, 2023; van Loon & Oeri, 2023).

Accurate monitoring of progress on a task is a prerequisite for effectively controlling learning activities and decisions (De Bruin & van Gog, 2012). However, assessing monitoring alone does not provide insight into how judgments guide actual decisions and actions during the learning process, making the inclusion of measures of control essential. Moreover, when addressing relations between metacognition and learning, the field has relied mainly on correlational methods (Muncer et al., 2022; Ohtani & Hisasaka, 2018), often using tasks for which learners cannot autonomously use their monitoring to control their learning. This limits understanding of the predictive effects of real‐time monitoring and control processes on learning outcomes. To address these gaps, the present study examines adolescents' real‐time monitoring and control processes to better understand their impact on learning outcomes during a cognitive task.

Metacognitive skills develop from early childhood through late adolescence, with age‐related improvements in monitoring one's cognitive performance and in using this monitoring to guide control decisions and actions (Moses‐Payne et al., 2021; Roebers, 2017; Schneider & Löffler, 2016). This study focuses on adolescence, a critical period when monitoring and control become increasingly vital for learning (van Loon & Oeri, 2023). As students enter secondary education, they must engage more in self‐regulated learning, an autonomous, proactive process involving continuous metacognitive monitoring and control to adjust behaviors and improve outcomes (Zimmerman, 2002). During adolescence, the refinement of metacognitive skills supports better decision‐making and enhances learning (Moses‐Payne et al., 2021; van Loon & Oeri, 2023). However, despite the importance of metacognition for adolescents' learning, procedural metacognitive processes during learning remain underresearched for this age group. The few existing studies indicate that although many adolescents show adaptive monitoring skills, there are notable individual differences in how these skills are translated into effective control decisions. For instance, van Loon and Roebers (2017) showed that at age 12, most students could (relatively) accurately monitor the quality of their self‐test responses. However, there was substantial variation in the effectiveness of their restudy decisions. Some adolescents decided to restudy all the task items they judged as not well learned and were likely to increase their knowledge, whereas others chose to restudy almost none of these and did thus not correct their mistakes.

In addition, most research on procedural metacognition relied on one‐time assessments. However, monitoring and control processes can vary between learning situations and change over time (Lehmann et al., 2022; van Loon et al., 2022). For instance, when completing a task for the first time, persons may use less refined strategies to monitor and control than when completing a task with which they have obtained experience and are familiar (Bayard et al., 2021; Paulus et al., 2014). That is, metacognitive development during adolescence is not solely age‐driven but is also shaped by experience with specific tasks.

The present study uses a repeated‐measures design to examine how adolescents' monitoring and control processes affect learning performance and how these metacognitive processes may shift over time with task experience. The task used in this study, where students learn a new language (Japanese Kanji) and make self‐monitoring judgments and restudy decisions, is presumed to provide insights into adolescents' broader metacognitive abilities. Previous research has shown that monitoring and control accuracy for this Kanji task is related to metacognitive skills in other tasks, such as monitoring and controlling text comprehension (van Loon et al., 2024). Thus, the task's measures likely reflect participants' skills to monitor and control their learning more generally. The research task is new to the participants at T1, whereas at the second time, they have experience with the task.

## PEER INFLUENCES ON METACOGNITION AND LEARNING

Even when working individually on learning tasks and engaging in metacognitive monitoring and control, these processes are still likely influenced by the surrounding social learning context (Hadwin et al., 2011). Social messages from parents, teachers, and peers appear to play a key role in metacognitive development. For example, parental metacognitive talk in preschool (e.g., prompting children to monitor and adapt strategies) predicts children's ability to monitor and control memory (Geurten & Léonard, 2023). In elementary school, the content of teachers' strategy instructions shapes children's restudy decisions and performance (van Loon et al., 2021). Although adults shape the social learning environment in childhood, friends gain importance during adolescence. Compared to children, adolescents have more freedom to interact with peers and are motivated by a strong desire to be accepted and liked by their friends. They value their friends' opinions and align their behavior with group norms (Bagwell & Bukowski, 2018; Wang et al., 2018). Friends influence a wide range of behaviors, including risk‐taking and delinquency (Andrews, 2020), as well as school attendance, learning effort, and academic achievement (Laninga‐Wijnen et al., 2019; Wang et al., 2018).

Peer similarity can emerge through both selection (choosing similar friends) and influence (becoming more similar over time through social learning, reinforcement, or shared experiences; Laninga‐Wijnen & Veenstra, 2021). Social network analyses help disentangle these processes. For social network studies investigating classroom friendship networks, findings show how adolescents select friends with similar levels of academic achievement (Laninga‐Wijnen et al., 2019; Rambaran et al., 2017) and also influence each other's behavioral engagement and achievement over time (Laninga‐Wijnen et al., 2019; Shin, 2018; Wang et al., 2018).

Selection and influence effects are most likely to occur for behaviors that are observable or discussed among friends, with processes like mutual encouragement contributing to friends becoming similar (Laninga‐Wijnen & Veenstra, 2021). For example, Wang et al. (2018) demonstrated peer influence on students' willingness to invest effort in school, suggesting that these engagement behaviors are visible to peers. One's metacognitive processes during learning may also be visible to others, and students may learn metacognitive skills by observing each other and through interaction. Paulus et al. (2014) showed that adolescents could infer others' metacognitive monitoring and control by observing how they allocated study time to easier or harder task items. Okita (2014) showed that observing a computer character solve problems and catch errors helped children improve their self‐monitoring and correct their errors. Moreover, friends may also influence each other through shared discussions about metacognitive processes and learning experiences. Adolescents have been shown to talk about school tasks and exchange information on confidence, decisions to review materials, and perceived task difficulty (Dindar et al., 2019; Hurme et al., 2006). These interactions may shape how students monitor and control their learning and how well they perform. Since on‐task metacognitive processes are partly observable (Paulus et al., 2014), discussed in peer conversations (Dindar et al., 2019; Hurme et al., 2006), and can improve through observation (Okita, 2014), possibly friends influence each other's metacognition. Over time, this may lead them to become more similar in how they learn.

However, despite evidence of selection and influence effects on general school achievement, little is known about which specific learning processes friends share that drive this similarity. The present study addresses this gap by examining how on‐task monitoring, control, and learning outcomes relate to classroom friendship networks. Specifically, we investigate whether friends show similarities in metacognitive processes, such as recognizing errors and making adaptive decisions. By combining on‐task measures with social network analyses across two time points, the study brings novel insights into how metacognition and learning are shaped by both individual experiences and social relationships. These findings contribute to theories of adolescent metacognitive development by highlighting how monitoring and control predict learning and how this may shift with task experience. This study further adds to theory about adolescents social development by showing to what extent metacognitive processes and task outcomes are embedded in, and shaped by classroom friendship networks.

## PRESENT STUDY

This study aims to explore the impact of metacognitive monitoring and control skills on learning scores, as well as the influence of friends on metacognition and learning. At two time points, 3 months apart, participants completed a metacognition task and indicated who their friends were in their classroom. For the metacognition task, they learned Japanese Kanjis as a novel language. They engaged in self‐testing, monitored their self‐test performance with confidence judgments, and controlled their learning by making restudy decisions and selecting which responses to submit for grading. Mimicking real‐life self‐regulated learning, they could allocate study time per item, choose what to restudy, decide when to stop learning new Kanjis, and opt out of submitting certain responses. Three on‐task components of metacognition were assessed: (a) monitoring accuracy (the extent to which confidence judgments align with actual self‐test performance), (b) monitoring‐based restudy (the extent to which restudy decisions are informed by monitoring judgments, such that items identified as not‐yet‐learned are prioritized), and (c) decision accuracy (the extent to which correct responses are submitted and incorrect responses are withdrawn, balancing the potential to earn or lose points). The 3‐month interval was chosen to capture short‐term changes in metacognition, task performance, and friendship networks. While this period is too brief to detect fine‐grained developmental progress, it allows insight into how participants' approaches to the metacognition task and their peer networks may shift over time.

The first aim is to examine the role of metacognitive skills on learning scores. We hypothesize that, at both time points, higher monitoring accuracy, monitoring‐based restudy, and decision accuracy will lead to higher task scores (H1). The second aim of this study is to examine peer influence on metacognitive skills and learning over time, while accounting for the possibility that adolescents with similar skills may be more likely to befriend each other (selection). Previous work found adolescents with similar academic behaviors (Wang et al., 2018) and achievement (e.g., Laninga‐Wijnen et al., 2019; Rambaran et al., 2017) to cluster together as friends, and we will investigate whether this also applies to adolescents' metacognitive skills. We therefore examine potential selection effects without formulating a specific hypothesis. By T2, the task is no longer new; participants gained experience with the metacognition task, may have observed how friends engaged with the task, or discussed the task with them, potentially affecting their own approach. We therefore hypothesize adolescents to become more similar to their friends in monitoring, control, and task performance from T1 to T2 (H2).

## MATERIALS AND METHODS

### Participants

Participants at T1 were 136 secondary school students (53.8% female; mean age 13.8 years) from eight seventh‐grade classrooms in secondary schools in Switzerland. At T2, 3 months later (beginning of eighth grade), 117 of them participated. Dropout was due to school changes, absences, or lack of motivation to complete the task the second time. Participants and their caretakers provided informed consent, with 80% to 90% of students having consent for data storage. Those without consent completed the metacognition task as a classroom activity, but their data was not stored, and they did not complete the social network questionnaire. Participation was voluntary, with the option to discontinue any time, contributing to some dropouts at T2. None of the participants had prior knowledge of Japanese Kanjis.

### Procedure

For both measurements, a researcher visited the class to introduce and administer the Kanji task. Participants with consent entered a unique participant number to start the task. After an introduction to the task phases (study, self‐test, monitoring, restudy, retest, response maintenance/withdrawal decisions) using two example Kanjis, they proceeded with the main task. After completing the Kanji task, the social network questionnaire about friendships was filled out. The procedure was identical at both time points. After both sessions, the class received money for their class fund as a reward for their participation.

### Materials and measures

#### Metacognition kanji task

**FIGURE 1:**
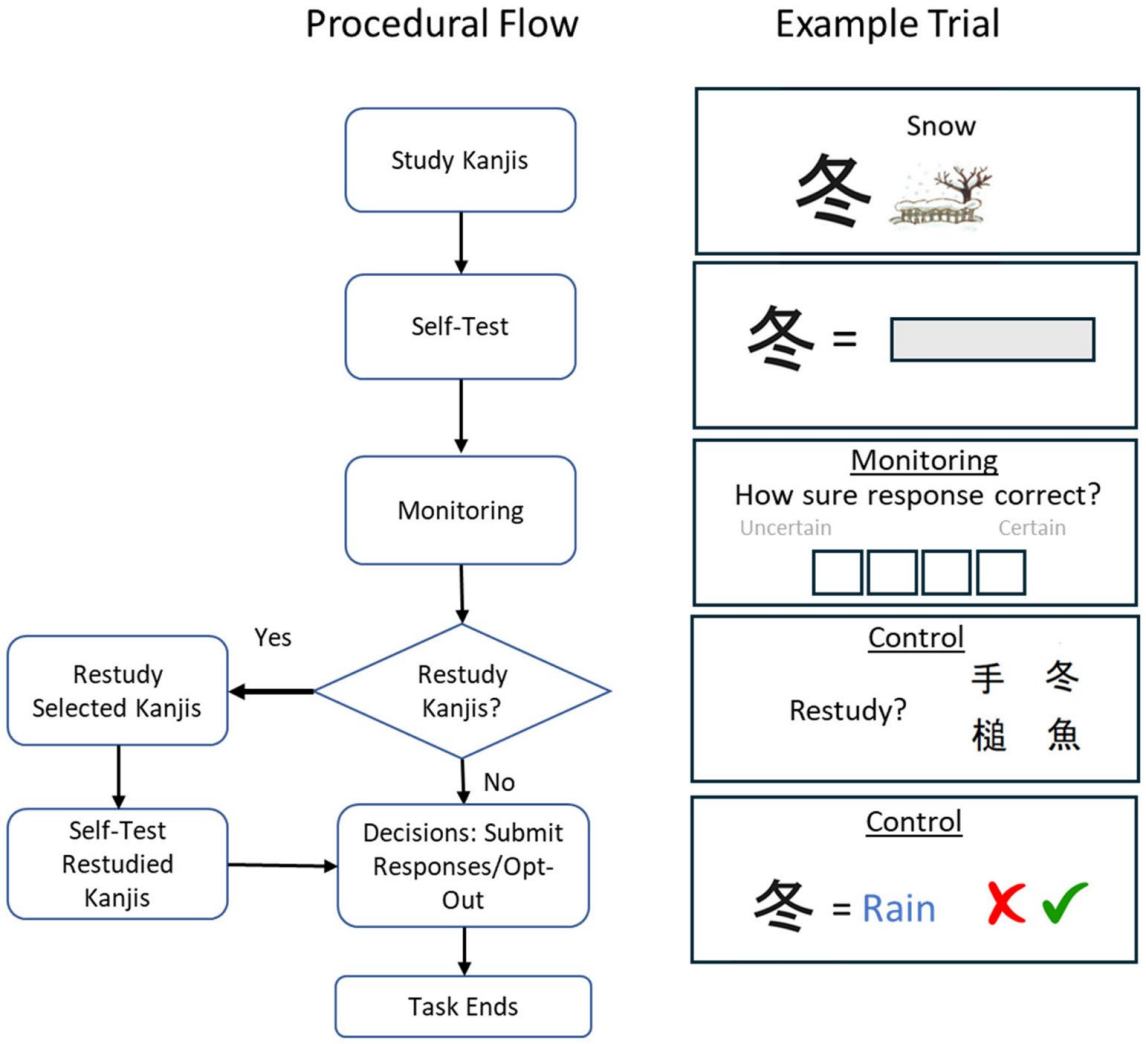
Procedural flow of the Kanji task.

The Kanji Task to assess metacognition was based on the procedure reported by van Loon and Oeri (2023), but with different materials (Kanjis instead of concepts). The Kanjis were previously used in studies on procedural metacognition by Destan and Roebers (2015) and Roebers et al. (2019). The task procedure was identical at both measurements, but different Kanjis were used. Kanji selections for both measurements were based on data from Roebers et al. (2019) to ensure comparable item difficulty for both sessions. The task was programmed in Qualtrics, with the procedural flow shown in Figure 1.

Participants learned 24 Kanjis across three blocks of 8, with a randomized item order within and between blocks. They studied at their own pace, followed by a self‐test after each block. After giving each test response, participants self‐monitored by rating their confidence in response correctness on a 4‐point scale. After completing the block, they could then control learning by deciding whether they liked to restudy some of the learned Kanjis. They saw the Kanjis (without meanings) in a grid and selected the ones they liked to restudy. After restudy, these selected items were retested, allowing participants to update their self‐test responses. After each block, participants decided whether to continue learning more Kanjis or to stop.

Finally, participants could control which self‐test responses to submit for grading. They saw all the studied Kanjis with their given responses about the meanings; for the restudied Kanjis, they saw the updated responses. They then decided, per the given response, if they would like to submit it or opt out and withdraw the response. Before making decisions, they were instructed that they would earn points for correct submissions while submitting incorrect answers would lead to point deductions.

For each Kanji item, test responses were rated 0 (incorrect) or 1 (correct), confidence judgments ranged from 0 to 3, restudy selections were coded as 0 (not selected) or 1 (selected), and accuracy of response maintenance/withdrawal decisions was coded as 1 (accurate) or 0 (inaccurate); making accurate decisions involved withdrawing incorrect responses and maintaining correct ones.

#### Social network questionnaire

At both measurement times, participants completed a social network questionnaire. This questionnaire was provided on paper (to enable adolescents to fold this and do this entirely anonymously). They were asked to look around in their class and report the names of their friends. They could select up to seven friends in their classroom and also decide to nominate no one. For the present study, nominations were limited to seven to capture a representative range of friendships while preventing outliers (e.g., students nominating all classmates as friends) and ensuring the questionnaire could be completed within the allotted testing time. This approach of restricting the number of nominations aligns with Wang et al. (2018), who, however, only allowed a maximum of three nominations. Shin and Ryan (2014) and Laninga‐Wijnen et al. (2019) allowed unlimited friend nominations and reported an average number of friends ranging between 4.20 and 6.59. A meta‐analysis by Neal (2025) found that when young adolescents could select an unlimited number of friends from a roster, they chose an average of 5.50. Thus, allowing up to seven nominations was expected to be sufficient for most students and to encourage more selective reporting of actual friendships. The friendship nominations were converted into one large adjacency matrix for all classrooms, involving nominators (in rows) and nominees (in columns), in which a ‘1’ indicates a friendship nomination, whereas a ‘0’ indicates no friendship nomination. Participants could only nominate friends within their own class, not from other classes in the study. These impossible ties were coded as 10, referred to as structural zeros, as required for Siena analysis (Ripley et al., 2024).

### Statistical analyses

#### Metacognition and task scores

To assess monitoring accuracy, we assessed how well participants' judgments (rated on a scale from 0 to 3) distinguished between correct and incorrect responses for the initial test (before restudy). Monitoring accuracy was calculated with the R package *lme4*, using linear mixed modeling following Murayama et al. (2014), to ensure it was independent of initial self‐test performance. We regressed item‐level initial performance on monitoring judgments, thereby investigating participants' ability to monitor performance, accounting for the nested data structure (items nested within participants) and individual differences in performance. Random intercepts were included to account for baseline differences in performance, and random slopes for performance allowed variation in how strongly self‐test performance predicted monitoring judgments across participants. Random slopes for each participant provided an individualized measure of monitoring accuracy, reflecting the extent to which initial performance was related to confidence judgments.

To assess monitoring‐based restudy, we calculated an intra‐individual measure by applying the linear mixed model approach suggested by Murayama et al. (2014) to regress monitoring judgments onto restudy decisions at the item level. In a subsequent step, we calculated random slopes per participant to calculate how strongly monitoring judgments were translated into study decisions.

Following Roebers and Spiess (2017), decision accuracy was calculated as the proportion of accurate decisions per participant. Task scores represented the total points participants earned for their submitted Kanji answers. To calculate these scores, all submitted answers were included, with the initial self‐test response for the not‐restudied Kanjis and the updated response used for Kanjis that were restudied and retested. Scores thus reflect participants' most recent test attempts.

#### Data imputation for social network analyses

For some participants, a part of the attribute data was missing (at T1 11.68%, at T2 14.6%) due to absences, participant dropouts, or technical difficulties during completion. Little's MCAR test showed that the missing attribute data were likely missing at random, *χ* ^2^(22) = 29.388, *p* = .134, indicating that imputation appeared suitable to deal with missings (van Ginkel et al., 2020). Multiple imputation was therefore used to impute missing student attribute data, using the Predictive Mean Matching (PMM) procedure (as recommended by van Ginkel et al., 2020). The variables selected as predictors in the PMM‐imputation model included Gender, Class number, and for both time points Mean Study Time, Initial Performance, Number of Kanjis learned, Monitoring Accuracy, Monitoring‐Based Restudy, Monitoring‐Based Decisions, Decision Accuracy, and Task Scores. Missing values were imputed five times per participant, and mean values for the imputed values were subsequently computed per participant.

Missing values in peer‐nomination data were 0.73% for T1 and 14.6% for T2. The method of moments (MoM) procedure used the model‐based hybrid imputation approach by Huisman and Steglich (2008) to handle missing tie variables. For T1, missing ties were imputed as zeros, reflecting the typical sparsity of social networks. For consecutive waves, missing ties were imputed using the last value carried forward (Lepkowski, 1986). Missing tie variables were excluded in the calculation of target statistics for parameter estimation.

#### Categorization of the metacognition and task score measures

The package RSiena is primarily designed to handle categorical attribute variables. Consistent with recommended practices in longitudinal social network analysis (Ripley et al., 2024), the continuous variable assessing monitoring accuracy, monitoring‐based restudy, and task scores was categorized into three bins (low, medium, and high) based on the distribution at T2. Decision accuracy was categorized into two bins (low and high) due to the large proportion of participants scoring at the highest level. With the model specifications, these categories were treated as having an ordinal nature. The categorization was based on the data distribution at the second time point (T2). To ensure comparability over time, we applied the same cutoff values to categorize the attribute variables for T1, ensuring that individual changes between categories over time reflected actual changes in underlying values, rather than time‐dependent thresholds. The online supplementary materials Data S1 provide further information about the categorization of the attribute variables and the binning cutoff values.

#### Hypothesis testing

For both time points (T1 and T2), the extent to which the metacognition components monitoring accuracy, monitoring‐based restudy, and decision accuracy predicted task scores was investigated with path analyses using the R‐package *lavaan* (Rosseel, 2012), testing Hypothesis 1. These analyses calculated the effects of the metacognition components on task scores while accounting for the correlations between them.

Longitudinal social network analyses were conducted to analyze whether friends influence adolescents' metacognition and task scores (Hypothesis 2) using the SIENA (Simulation Investigation for Empirical Network Analysis) method with the *rsiena* package (Snijders et al., 2010, available at https://www.stats.ox.ac.uk/~snijders/siena/). These analyses examined the dynamic co‐evolution of peer relationships and metacognition, testing for selection effects (i.e., whether similarities in metacognition and performance predict the formation or maintenance of peer ties) and influence (i.e., whether adolescents become more similar in metacognition and task scores to their friends over time). Separate models for each attribute (monitoring accuracy, monitoring‐based restudy, decision accuracy, and task scores) were estimated. Structural network effects were included to control for natural dynamics within the friendship network, including reciprocity (the tendency to reciprocate friendships), the tendency of students to become friends of their friends (geometrically weighted edgewise shared partners; gwespFF), squared indegree popularity (students with many friendship nominations receive even more friendships over time), and squared outdegree activity (students who send out many friendship nominations tend to send out more friendship ties over time). Gender‐related effects (EgoX, AltX, and sameX for Gender) controlled for the influence of gender on friendship dynamics. To model friendship selection effects related to metacognition and task scores, EgoX and AltX effects modeled the role of attributes in the number of outgoing and incoming friendship ties, respectively. The simX term was included to investigate selection effects, i.e., whether adolescents who are more similar in a specific attribute are more likely to become friends over time. The avSim parameter was used to estimate influence effects, i.e., the tendency for adolescents to become more similar to their friends over time. We controlled for the general development of metacognitive skills and performance across the two time points (linear and quadratic shape) and for the extent to which a high incoming (indeg) or outgoing (outdeg) number of friendships related to a stronger increase in metacognition and task scores.

Convergence and goodness of fit were assessed using the criteria from Ripley et al. (2024). All *t*‐ratios for convergence were <0.10, and the overall maximum convergence ratio was <0.25. Goodness of fit was evaluated by simulating 3000 networks and comparing them to the observed data for indegree and outdegree distributions, geodesic distances, triad census, and behavioral distributions. The models fitted well in terms of indegree, outdegree, geodesic distance, and behavioral distributions. Although goodness of fit significance tests were significant for the triad census, violin plots indicated that most triad types were well represented (see Data S1).

### Transparency, IRB, and data availability statement

The study design, hypotheses, and analyses were preregistered prior to data collection, link: https://osf.io/kqvgu/. The study design was approved by the ethical review committee of the Faculty of Human Sciences at the University of Bern, Switzerland. More hypotheses were preregistered than tested in this manuscript. While we originally planned to examine correlations between metacognition indicators within and across time points, these are reported as descriptive statistics in line with the preregistered analysis plan but are not the focus of this paper. Similarly, persistence was excluded from analyses due to ceiling effects, as most participants completed the task.

Minor wording changes were made for Hypothesis 1 (preregistered as Hypothesis 2). For Hypothesis 2 (preregistered as Hypothesis 4), the original hypothesis on influence effects was that “changes in metacognition over time are related to changes in the peer network.” The preregistered analyses, however, included plans to test both selection and influence effects.

In line with the preregistration, we aimed to recruit six school classes, targeting at least 100 participants. To account for potential dropout over time, we oversampled at T1 and recruited eight school classes. The attribute and social network data sets, analysis scripts, and Data are available online here: https://osf.io/kqvgu/.

## RESULTS

### Descriptive statistics and preliminary analyses

Before testing hypotheses, we present descriptive statistics for monitoring, control, and task scores, including T1 to T2 comparisons. As preregistered, we report correlations between monitoring accuracy, restudy decisions, decision accuracy, and task scores for time points separately, and Pearson correlations across T1 and T2. Further, descriptive statistics for social friendship networks are included.

#### Metacognition and task scores

Table 1 presents the means, standard deviations, and *t*‐test statistics comparing the values for T1 and T2 for monitoring, control, and task scores. Calculations of monitoring accuracy indicated that self‐test performance predicted confidence judgments at T1 (Std. Est. = 0.70, SE = 0.02, *t*(2571) = 33.66, *p* < .001) and T2 (Std. Est. = 0.71, SE = 0.025, *t*(2557) = 28.24, *p* < .001), indicating high monitoring at both times. There were no differences in monitoring accuracy between T1 and T2, *Z* = −0.25, *p* = .81. Monitoring at T1 was a significant predictor of restudy decisions (Std. Est. = −0.44, SE = 0.03, *t*(2571) = −13.55, *p* < .001), indicating that higher judgments were less often translated into restudy decisions than lower judgments; the coefficient indicates a moderately strong relation. Also for T2, monitoring was a negative predictor of restudy (Std. Est. = −0.46, SE = 0.04, *t*(2540) = −12.56, *p* < .001). The monitoring‐based restudy coefficients did not differ between T1 and T2, *Z* = 0.38, *p* = .70.

**TABLE 1:** Means, standard deviations, and *t*‐test statistics comparing the values for T1 and T2 for monitoring, control, and task scores.

Measure	T1 mean (SD)	T2 mean (SD)	*T‐*value and significance
Proportion correct initial self‐test responses	0.70 (0.23)	72 (0.25)	−0.517, *p* = .606
Mean confidence judgments (range 0–3)	1.86 (0.63)	1.99 (0.71)	−2.090, *p* = .039
Mean confidence correct self‐test responses	2.39 (0.50)	2.52 (0.47)	−2.427, *p* = .017
Mean confidence incorrect self‐test responses	0.51 (0.45)	0.56 (0.65)	−0.639, *p* = .525
Mean restudy proportion	0.46 (0.31)	0.36 (0.32)	3.343, *p* = .001
Restudy proportion when self‐test response correct	0.38 (0.32)	0.27 (0.31)	3.330, *p* = .001
Restudy proportion when self‐test response incorrect	0.78 (0.33)	0.64 (0.42)	3.414, *p* < .001
Proportion maintained correct responses	0.81 (0.23)	0.91 (0.13)	−2.338, *p* = .022
Proportion maintained incorrect responses	0.17 (0.30)	0.35 (0.41)	−1.810, *p* = .081
Task scores (range 0–24)	17.06 (7.03)	16.41 (9.56)	0.622, *p* = .536

*Note*: *T*‐test statistics are reported for paired two‐sided *t*‐tests.

##### Correlations between components of metacognition

Table 2 shows the correlations between metacognition components and task scores at both time points. At T1, monitoring accuracy and decision accuracy were moderately positively correlated. Additionally, at T1, monitoring accuracy was moderately correlated, and decision accuracy was strongly correlated with task scores. At T2, all metacognitive components were significantly correlated. Note that for monitoring‐based restudy, negative values indicate more effective restudy (i.e., restudy is more frequent for items with low confidence judgments than for those with high confidence judgments). The weak negative correlation suggests that higher monitoring accuracy was related to more effective monitoring‐based restudy. Further, monitoring accuracy was weakly to moderately positively correlated with decision accuracy, and more effective monitoring‐based restudy was moderately correlated with higher decision accuracy. Finally, at T2, all metacognitive components showed moderate to strong correlations with task scores.

**TABLE 2:** Correlations between metacognition measures and task scores.

Measure	Monitoring accuracy	Monitoring‐based‐restudy	Decision accuracy	Task scores
Monitoring accuracy	—	−0.091	0.348**	0.305**
Monitoring‐based‐restudy	−0.188*	—	−0.063	−0.120
Decision accuracy	0.218*	−0.348**	‐	0.711**
Task scores	0.271**	−0.515**	0.658**	—

*Note*: Pearson correlations between the metacognition variables and task scores at T1 (above the diagonal) and T2 (below the diagonal).

* *p* < .05.

** *p* < .001.

##### Stability of metacognition and task scores over time

For the metacognition components and task scores, Pearson correlations were calculated between T1 and T2 to investigate the rank‐order stability of these measures over time. Monitoring accuracy measures for T1 and T2 were significantly but weakly correlated, *r* = .189, *p* = .048. The correlations between T1 and T2 were significant and moderate for monitoring‐based restudy, *r* = .426, *p* < .001; decision accuracy, *r* = .349, *p* < .001; and task scores, *r* =.293, *p* = .004.

##### Social friendship networks

**FIGURE 2:**
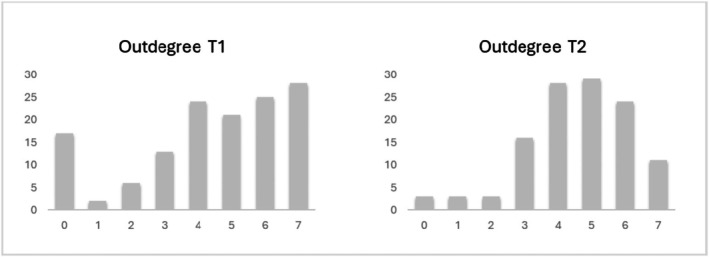
Outdegree distribution at T1 and T2. Participants could nominate up to 7 classmates as friends. The figure shows the frequency distribution of the number of friendship nominations made.

At T1, participants indicated that they had, on average, 4.38 friends in their class; for T2, they indicated an average of 3.91 friends. Figure 2 shows the frequency distribution of the number of friendship nominations made for both time points. Of the friendships, 74% were reciprocated at T1 and 77% at T2. The friendships were sufficiently stable over time to conduct social network analyses (Jaccard Index = 0.63).

Moran's *I* was weakly negative for all constructs at both time points for metacognition and task scores. Specifically, at T1, Moran's *I* was −0.01 for monitoring accuracy, −0.03 for monitoring‐based restudy, −0.01 for decision accuracy, and −0.01 for task scores. For T2, Moran's *I* was −0.03 for monitoring accuracy, −0.03 for monitoring‐based restudy, −0.01 for decision accuracy, and −0.02 for task scores. These values suggest that, on average, friends were not very similar in metacognition and task scores.

### Effects of metacognition on task scores

Path analyses investigated whether monitoring accuracy, monitoring‐based restudy, and decision accuracy predict task scores. Table 3 shows the unstandardized and standardized path coefficients for the path models for T1 and T2. As indicated by Table 3, at T1, only decision accuracy predicted task scores; the standardized estimate indicates this effect was strong. There were no significant effects of monitoring and monitoring‐based restudy on task scores at T1. At T2, both monitoring‐based restudy and decision accuracy significantly predicted task scores. Note that, for monitoring‐based restudy, the direction of the effect (i.e., a negative effect) indicates that more consistently translating lower rather than higher confidence judgments into restudy led to higher scores. There was no effect of monitoring accuracy on task scores. This partially confirms Hypothesis 1: only control components, but not monitoring accuracy, did predict task scores.

**TABLE 3:** Effects of metacognition on task scores at T1 and T2.

Predictors of task scores	T1			T2		
	Estimate (unstandardized)	Standard error	Estimate (standardized)	Estimate (unstandardized)	Standard error	Estimate (standardized)
Monitoring accuracy	1.589	1.594	0.07	2.14	1.441	0.09
Monitoring‐based‐restudy	−5.095	4.622	−0.08	−27.518**	5.954	0.31**
Decision accuracy	20.537**	2.188	0.68**	23.429**	3.03	0.53**
Explained variance (*R* ^2^)	0.48			0.47		

*Note*: Path models investigating effects of the components of metacognition on task scores for T1 and T2.

** *p* < .01.

### Social network analyses

#### Friendship influence on metacognition and task scores

Table 4 shows the estimated model parameters from the social network analyses. These estimates reflect changes in both the network structure and the attribute variables across T1 and T2, as they are derived from data spanning both time points to model the dynamic processes of selection and influence. In contrast to our hypothesis (H2), friends had no significant influence on adolescents' metacognitive skills (monitoring accuracy, monitoring‐based restudy, and decision accuracy; Table 4). There was, however, a significant friendship influence effect on task scores (Est. = 4.473, SE = 2.087), suggesting that friends became more similar (or less dissimilar) over time in how they scored on the task.

**TABLE 4:** Model parameters for the longitudinal social network analyses for the metacognition and task score attributes.

Attributes	Monitoring accuracy		Monitoring‐based‐restudy		Decision accuracy		Task scores	
Parameters:	Estimate	SE	Estimate	SE	Estimate	SE	Estimate	SE
Rate parameter friendships	3.754**	0.881	4.675**	0.522	4.621**	0.499	4.597**	0.465
Outdegree (density)	7.477	9.863	4.927*	2.201	3.915*	1.438	4.735	2.47
Reciprocity	1.902	1.702	1.53**	0.251	1.414**	0.246	1.647**	0.394
Triadic relationships (GWESP)	3.623	3.335	2.486**	0.456	2.395**	0.374	2.475**	0.431
Indegree – popularity (sqrt)	−2.58	3.497	−1.378**	0.359	−1.395**	0.356	−1.4**	0.341
Outdegree – activity (sqrt)	−2.122	2.042	−1.714*	0.635	−1.338**	0.352	−1.632*	0.714
Gender alter	−0.421	1.124	−0.167	0.206	−0.149	0.187	−0.172	0.204
Gender ego	0.074	0.42	−0.108	0.281	−0.061	0.224	−0.127	0.361
Gender selection similarity	−0.133	0.311	−0.019	0.204	−0.079	0.197	−0.147	0.218
Attribute alter	0.987	2.863	−0.146	0.189	0.457	0.354	0.427	0.272
Attribute ego	−0.282	1.032	0.892*	0.421	−0.777	0.473	−1.004	0.701
Attribute similarity (Selection)	4.869	7.610	0.6	0.763	−0.421	0.464	0.047	0.947
Rate attribute	4.678*	1.630	2.621*	0.599	0.939*	0.337	3.126**	0.842
Attribute linear shape	−0.443	0.370	−0.414	0.49	−5.068	12.193	−0.432	0.478
Attribute quadratic shape	0.409	0.269	0.426	0.258			0.461*	0.226
Attribute average similarity (Influence)	−0.021	0.889	0.153	0.721	1.34	3.557	2.119*	0.757
Attribute indegree	0.100	0.125	0.078	0.125	−0.919	3.131	−0.018	0.135
Attribute outdegree	−0.011	0.149	0.001	0.15	1.914	5.474	0.078	0.177

*Note*: Estimates reflect the parameter estimates from the models calculated for four attributes: monitoring accuracy, monitoring‐based restudy, decision accuracy, and task scores. Each attribute was modeled separately, with estimates for selection effects (attribute alter, ego, and similarity) and influence effects (average similarity). Quadratic shapes were not estimated for decision accuracy, as this attribute variable was categorized into two bins (low/high). Significance levels are indicated with *p < .05 and **p < .01.

#### Additional effects

To reliably estimate the influence of friends on metacognitive skills and task scores, we controlled for the extent to which adolescents selected (or maintained) friends based on pre‐existing similarities in these attributes. None of the similarity‐based selection effects were significant, suggesting that adolescents do not befriend peers based on similarity in metacognitive skills or task performance. There were only two effects of metacognition and task performance on network tendencies: adolescents high in monitoring‐based restudy and those low in task performance were more likely to send out friendship nominations.

Other effects in the model indicate that adolescents reciprocated their friendships, became friends with the friends of their friends, and that those receiving or giving more nominations received or gave more nominations over time. The positive quadratic term for task scores suggests that adolescents with low or high task scores at T1 were more likely to stay in their respective categories; adolescents with medium scores were more likely to switch task score categories (either shifting to high or low task scores). Moreover, adolescents who sent out or received many friendship nominations were not more likely to increase in metacognitive skills or task scores, as indicated by non‐significant attribute outdegree and indegree estimates.

## DISCUSSION

Adolescents' metacognitive skills and social relationships play key roles in learning but are often studied in isolation. This study investigated the links between metacognition, learning performance, and classroom friendship networks across two time points 3 months apart. Specifically, the first aim was to examine how monitoring accuracy, monitoring‐based restudy, and decision accuracy predicted task performance scores. Moreover, the second aim was to examine, using social network analyses, the influence of friends on adolescents' metacognition and task scores over time.

### Effects of metacognition on task scores

Decision accuracy emerged as the strongest predictor of task scores at both time points, while monitoring‐based restudy predicted performance only at T2. Interestingly, participants restudied fewer items overall at T2 as compared to at T1, particularly reducing restudy of incorrectly answered items. This may reflect that, with task experience, they became more selective in allocating their effort and resources. At the same time, despite becoming more selective, the stronger effect of monitoring‐based restudy on task scores at T2 suggests that task experience enhanced the effectiveness of restudy decisions.

The “Dual Mechanisms of Control Framework” (Braver, 2012) may support with interpreting these shifts over time. Monitoring‐based restudy appears a form of proactive control; participants take forward‐looking, goal‐directed actions to improve their performance through restudy. In contrast, decision accuracy may reflect reactive control, a late correction mechanism used just in time to address immediate demands (i.e., dealing with a risk of losing points). At T1, when the task was unfamiliar, reactive control (decision accuracy) was the key predictor of task scores. By T2, proactive control (strategic restudy) became an additional predictor. This suggests that with task experience, adolescents could better balance proactive strategies with reactive strategies to minimize errors and enhance their scores. Research indicates a developmental shift from reactive to proactive control between childhood and adulthood (Niebaum et al., 2021). The findings of this study suggest that for adolescents who autonomously work on cognitive learning tasks, task experience may foster a shift from reliance on reactive control to integrating both proactive and reactive strategies.

Adolescents consistently used their monitoring judgments to guide their decisions. While correlational analyses indicated that monitoring accuracy was related to task scores, path analyses showed that decision accuracy predicted performance, rather than monitoring accuracy. This indicates that monitoring accuracy alone did not drive task outcomes; instead, actual control appeared to mediate the relation between monitoring and task performance. These findings highlight the importance of examining not only the accuracy of monitoring judgments but also the quality of the control actions they inform. If this study had relied solely on correlations, the conclusions about the role of monitoring might have been different. Path analyses enabled uncovering the directional effects of control, demonstrating that the translation of monitoring into effective control actions is critical for performance.

### Social influences on metacognition and task scores

From T1 to T2, adolescents adapted their approach to the task: they became more confident in correct responses and engaged in less overall restudy. Our second aim was to understand whether and how social processes may influence adolescents' metacognition and learning over time. Given the classroom setting, observation of and social interactions with classroom friends were presumed to play a role. Although participants worked individually on the computer, they were in a classroom setting where they could see their peers. They may have observed how their friends approached the task. After the first session, they might also have discussed the task's value, their strategies, and the effort they invested. Such observations and exchanges could have influenced participants' decisions to invest effort, choose what to restudy, or take risks when submitting responses.

Using longitudinal social network analyses, we examined how friendships related to metacognition and task performance. Our findings showed that while friends did not influence each other's metacognitive processes, they did become more similar in task scores over time. These findings are consistent with prior research on peer influence in academic achievement (e.g., Shin & Ryan, 2014; Wang et al., 2018). However, prior research demonstrating friendship influences on achievement has mainly relied on report card measures such as GPAs, teacher ratings, and self‐reported grades, which may introduce subjectivity bias. For example, teacher ratings can be affected not only by academic performance but also by a student's social behavior or teachers' beliefs about their abilities (Vosylis et al., 2024). The present study adds to this by showing that peer influence can also be observed in objectively measured task scores.

We did not find evidence for selection effects, meaning that students did not choose friends based on similar task scores. This might be due to the nature of the task, a novel and unfamiliar Japanese Kanji learning activity. The task did not count for school grades and may thus not have been perceived as a common school learning task and may therefore not be reflective of more general academic ability. However, the findings show influence effects for task scores, indicating that friends became more similar in their task performance over time. The present study is the first to find that friends influenced task scores over time and became more similar in performance on a specific research task designed to objectively measure learning processes and task scores on the item level. These findings suggest that peer influence extends beyond general academic achievement.

Of course, adolescents' performance is not a behavioral process by itself, but the outcome of underlying processes, attitudes, and actions that influence task scores. Although metacognitive processes were predictive of individual task performance, we did not find that friends influenced each other's metacognitive processes. That is, the observed influence of friends on task scores is not explained by direct peer influence on monitoring or control. It therefore remains an open question which specific attitudes and behaviors were influenced by friends. The task was designed as a self‐regulated learning (SRL) activity, allowing participants to decide autonomously how long to study, what to restudy, when to stop, and what to submit for scoring. Our metacognition measures were collected during the performance phase of SRL, that is, while working on the task. However, SRL is a cyclical process that includes forethought (goal setting and planning), performance, and reflection (evaluating plans and strategies; Zimmerman, 2002). The absence of peer influence on metacognition may indicate that the measured monitoring and control processes during the performance phase are more individual than socially driven. Future studies could examine whether and how social influences shape processes during forethought (e.g., goal‐setting) and reflection phases.

Moreover, SRL involves not only metacognition but also motivation and affect (Zimmerman, 2002). Motivation may be more visible, discussed, and socially influenced than metacognition. Although we did not measure motivation directly, behavioral changes over time, such as reduced restudy and fewer corrected errors at T2, might reflect shifts in motivation. To better understand peer influence on SRL and explain increasing similarity in performance, future research should broaden the focus to include the full SRL cycle and also incorporate measures of motivation.

We found that friends influenced task scores such that they became more similar over time in Kanji task performance; however, the current data do not allow us to directly examine the mechanisms behind these influence effects. Prior work points to several potential active and passive influence processes (Laninga‐Wijnen & Veenstra, 2021). For instance, active processes such as mutual encouragement (e.g., exchanging ideas, motivating each other to perform well) or co‐rumination (e.g., dwelling on the difficulty or low value of the task) may have contributed to friends becoming more similar. Active interactions likely occur, as adolescents exchange messages about their learning processes (Dindar et al., 2019). Although we did not assess interactions, it is plausible that friends shared experiences and ideas about the task, influencing each other's learning and outcomes. Moreover, passive influence mechanisms may play a role such as, for instance, imitation (Laninga‐Wijnen & Veenstra, 2021). Adolescents may have observed and copied friends' behaviors, such as actively focusing on the computer screen during Kanji learning or, conversely, disengaging by slouching and looking away. Further, norm conformity, where students align with perceived classroom expectations about effort and engagement, may explain influence effects. For instance, adolescents may have conformed to norms within their friendship group that discouraged or encouraged scoring high on the task. Previous work has shown that in adolescence, especially popular peers may set a norm. In some peer groups, working hard and doing well in school fits the norm and can increase social status (Laninga‐Wijnen et al., 2019). In other groups, the opposite is true; not working hard or performing well fits the norm better (Schwartz et al., 2013). So, how well adolescents learned in this study may have been influenced by their friends' group norms and whether being a good student was related to having higher or lower social status. In sum, it is likely that multiple active and passive peer influence processes contributed to friends becoming more similar in scores. These mechanisms are not mutually exclusive and may interact in complex ways. Future research should explore how social dynamics shape learning by examining peer interactions more closely.

Our findings contribute to theories of adolescent metacognitive development by showing that it is not monitoring accuracy per se, but rather how monitoring is used to guide control that drives learning outcomes. Within a short time frame and after just one experience with the task, adolescents' metacognitive control became more strategic and more predictive of performance. This suggests that task experience can enhance fine‐grained, proactive, and goal‐directed control. Moreover, our findings advance understanding of adolescents' social development by showing that individual changes in learning and performance are embedded in and shaped by the social context of classroom friendships. The results suggest that, during early adolescence, metacognitive monitoring and control may develop primarily through individual experiences, whereas learning performance is shaped by the social environment, for instance through mutual encouragement or peer imitation. Together, these findings underscore the importance of considering both intra‐individual development and interpersonal influences when studying how adolescents learn and perform in classroom settings.

### Limitations and future directions

While the strengths of our longitudinal design and the focus on adolescents provide valuable insights, several limitations should be considered. Firstly, limited statistical power may have prevented us from detecting influence effects. For longitudinal social network analysis, no established guidelines exist for required sample sizes, but key factors affecting power include network size, number of measurement points, and missing data (Stadtfeld et al., 2020). This study used only two measurement points, potentially limiting power, although low participant turnover and the use of imputation to address missing data may have mitigated this issue. Snijders et al. (2010) suggest that networks should include at least 20 actors and a minimum of 40 observed tie changes for reliable parameter estimation. Our dataset included 232 friendship changes, which may indicate that the sample and network data were sufficient for the analyses. All models converged and showed good fit; however, this does not guarantee sufficient power. Future research should therefore examine peer influences on adolescents' metacognitive skills using larger samples and additional measurement points.

Monitoring accuracy was notably high in this study, likely due to the recall‐based task and delayed self‐testing (Dunlosky et al., 2016), rather than reflecting generally strong self‐monitoring skills. This high accuracy reduced variability, which may explain why monitoring was less predictive of performance and less stable over time compared to control and task scores. Moreover, many participants had high decision accuracy and task performance, suggesting that the task may have overestimated adolescents' metacognitive abilities. These high scores may not fully reflect monitoring in more complex learning situations. The research task, an individual self‐study activity to memorize Kanji characters, reflects a low level of cognitive processing (Anderson & Krathwohl, 2001), and for such tasks, influence from peer interactions or classroom friendships may have been limited. More complex reasoning and problem‐solving tasks, especially those involving collaboration, are more likely to stimulate peer discussion and interaction, potentially making social influences on metacognition more visible. In such settings, peer exchanges may shape not only performance but also metacognitive processes. Future research should therefore use higher‐order cognitive tasks and collaborative learning contexts to better investigate whether and how social influences affect metacognition.

Another limitation is our reliance on classroom‐based friendship networks. This focus excluded broader social influences, such as the home learning environment, which may also shape adolescents' metacognition and learning. Moreover, while classroom friendships are a valuable social construct and help explain processes related to academic achievement (Laninga‐Wijnen & Veenstra,^2021^), they may not reflect the more specific peer dynamics relevant for metacognitive processes. Friendships in classrooms provide a general social context for interaction, but they may lack the specificity needed to capture peer influence effects on fine‐grained, on‐task metacognitive processes. Peer‐related learning constructs such as peer learning strategies, collaborative learning, and peer feedback may offer more direct pathways to activating and influencing metacognitive monitoring and control skills. Future research could focus on peer‐learning networks and interactions, rather than general classroom friendships, to better understand how social peer learning dynamics influence metacognitive development.

## CONCLUSIONS

This study advances our understanding of metacognition during adolescence by integrating individual and social perspectives. The findings highlight that individual on‐task metacognitive control skills and social factors influence adolescents' task scores. Future research could build on these findings to address individual differences in metacognitive skills. Findings may fuel intervention research by indicating that, rather than focusing solely on monitoring accuracy, interventions aimed at improving learning outcomes may be more effective when they emphasize the effective translation of monitoring into strategic control processes, such as restudy and learning decisions. Moreover, this study provides novel insights into the influence of social contexts on adolescents' learning. Findings show that classroom friendship relations influenced real‐time performance outcomes at the individual task level. These findings underscore the need to account for social contexts when aiming to understand and enhance adolescents' learning.

Encouraging adaptive metacognitive control and fostering positive peer interactions may offer two promising pathways for improving adolescents' learning outcomes. Future research should extend this work with the use of more complex, real‐world learning tasks to improve insights into how social dynamics shape learning processes.

## AUTHOR CONTRIBUTIONS

**Mariëtte van Loon:** Conceptualization; methodology; data curation; investigation; formal analysis; funding acquisition; project administration; writing – original draft; writing – review and editing. **Lydia Laninga‐Wijnen:** Methodology; formal analysis; writing – original draft; writing – review and editing.

## FUNDING INFORMATION

This research was supported by the Schweizerischer Nationalfonds zur Fördering der Wissenschaftlichen Forschung/Swiss National Science Foundation (SNSF), Grant Number TMSGI1_211411.

## CONFLICT OF INTEREST STATEMENT

No conflicts of interest exist.

## ETHICS STATEMENT

This study was approved by the Institutional Review Board of the University of Bern, Faculty of Human Sciences, on June 9, 2022 (Reference Number: 2022–06‐00002).

## CONSENT STATEMENT

Parent/guardian consent was obtained before testing for the research participants.

## Supporting information


Data S1

## Data Availability

The data that support the findings of this study are openly available in OSF at https://osf.io/kqvgu/.

## References

[jora70072-bib-0001] Anderson, L. W. , & Krathwohl, D. R. (2001). A taxonomy for learning, teaching, and assessing: A revision of Bloom's taxonomy of educational objectives. Allyn & Bacon (Pearson Education Group).

[jora70072-bib-0002] Andrews, N. C. Z. (2020). Prestigious youth are leaders, but central youth are powerful: What social network position tells us about peer relationships. Journal of Youth and Adolescence, 49(3), 631–644. 10.1007/s10964-019-01080-5 31301026

[jora70072-bib-0003] Baars, M. (2023). Understanding and supporting metacognitive monitoring judgments—A commentary. Zeitschrift für Entwicklungspsychologie Und Pädagogische Psychologie, 55, 147–154. 10.1026/0049-8637/a000282

[jora70072-bib-0004] Bagwell, C. L. , & Bukowski, W. M. (2018). Friendship in childhood and adolescence: Features, effects, and processes. In W. M. Bukowski , B. Laursen , & K. H. Rubin (Eds.), Handbook of peer interactions, relationships, and groups (2nd ed., pp. 371–390). The Guilford Press.

[jora70072-bib-0005] Bayard, N. S. , van Loon, M. H. , Steiner, M. , & Roebers, C. M. (2021). Developmental improvements and persisting difficulties in children's metacognitive monitoring and control skills: Cross‐sectional and longitudinal perspectives. Child Development, 92(3), 1118–1136. 10.1111/cdev.13486 33529372 PMC8248442

[jora70072-bib-0006] Braver, T. S. (2012). The variable nature of cognitive control: A dual mechanisms framework. Trends in Cognitive Sciences, 16(2), 106–113. 10.1016/j.tics.2011.12.010 22245618 PMC3289517

[jora70072-bib-0007] De Bruin, A. B. H. , & van Gog, T. (2012). Improving self‐monitoring and self‐regulation: From cognitive psychology to the classroom. Learning and Instruction, 22(4), 245–252. 10.1016/j.learninstruc.2012.01.003

[jora70072-bib-0008] Destan, N. , & Roebers, C. M. (2015). What are the metacognitive costs of young children's overconfidence? Metacognition and Learning, 10(3), 347–374. 10.1007/s11409-014-9133-z

[jora70072-bib-0009] Dindar, M. , Alikhani, I. , Malmberg, J. , Järvelä, S. , & Seppänen, T. (2019). Examining shared monitoring in collaborative learning: A case of a recurrence quantification analysis approach. Computers in Human Behavior, 100, 335–344. 10.1016/j.chb.2019.03.004

[jora70072-bib-0010] Dunlosky, J. , Mueller, M. L. , & Thiede, K. W. (2016). Methodology for investigating human metamemory: Problems and pitfalls. In J. Dunlosky & S. U. K. Tauber (Eds.), The Oxford handbook of metamemory (pp. 23–38). Oxford University Press. 10.1093/oxfordhb/9780199336746.013.14

[jora70072-bib-0011] Gascoine, L. , Higgins, S. , & Wall, K. (2017). The assessment of metacognition in children aged 4–16 years: A systematic review. Review of Education, 5(1), 3–57. 10.1002/rev3.3077

[jora70072-bib-0012] Geurten, M. , & Léonard, C. (2023). Relations between parental metacognitive talk and children's early metacognition and memory. Journal of Experimental Child Psychology, 226, 105577. 10.1016/j.jecp.2022.105577 36335835

[jora70072-bib-0013] Hadwin, A. F. , Järvelä, S. , & Miller, M. (2011). Self‐regulated, co‐regulated, and socially shared regulation of learning. In B. J. Zimmerman & D. H. Schunk (Eds.), Handbook of self‐regulation of learning and performance (pp. 65–84). Routledge/Taylor & Francis Group.

[jora70072-bib-0014] Huisman, M. , & Steglich, C. (2008). Treatment of non‐response in longitudinal network studies. Social Networks, 30(4), 297–308. 10.1016/j.socnet.2008.04.004

[jora70072-bib-0015] Hurme, T.‐R. , Palonen, T. , & Järvelä, S. (2006). Metacognition in joint discussions: An analysis of the patterns of interaction and the metacognitive content of the networked discussions in mathematics. Metacognition and Learning, 1(2), 181–200. 10.1007/s11409-006-9792-5

[jora70072-bib-0016] Laninga‐Wijnen, L. , Gremmen, M. C. , Dijkstra, J. K. , Veenstra, R. , Vollebergh, W. A. M. , & Harakeh, Z. (2019). The role of academic status norms in friendship selection and influence processes related to academic achievement. Developmental Psychology, 55(2), 337–350. 10.1037/dev0000611 30550323

[jora70072-bib-0017] Laninga‐Wijnen, L. , & Veenstra, R. (2021). Peer similarity in adolescent social networks: Types of selection and influence, and factors contributing to openness to peer influence. In B. Halpern‐Felsher (Ed.), Encyclopedia of child and adolescent health (pp. 196–206). Elsevier. 10.1016/B978-0-12-818872-9.00059-5

[jora70072-bib-0018] Lehmann, M. , Hagen, J. , & Ettinger, U. (2022). Unity and diversity of metacognition. Journal of Experimental Psychology: General, 151(10), 2396–2417. 10.1037/xge0001197 35389740

[jora70072-bib-0019] Lepkowski, J. (1986). The treatment of wave nonresponse in panel surveys. In G. Kalton , J. Lepkowski , S. Heeringa , T.‐T. Lin , & M. E. Mille (Eds.), The survey of income and program participation. The treatment of person‐wave nonresponse in longitudinal surveys (pp. 90–127). U.S. Department of Commerce Bureau of the Census.

[jora70072-bib-0020] Moses‐Payne, M. E. , Habicht, J. , Bowler, A. , Steinbeis, N. , & Hauser, T. U. (2021). I know better! Emerging metacognition allows adolescents to ignore false advice. Developmental Science, 24(5), e13101. 10.1111/desc.13101 33686737 PMC8612133

[jora70072-bib-0021] Muncer, G. , Higham, P. A. , Gosling, C. J. , Cortese, S. , Wood‐Downie, H. , & Hadwin, J. A. (2022). A meta‐analysis investigating the association between metacognition and math performance in adolescence. Educational Psychology Review, 34(1), 301–334. 10.1007/s10648-021-09620-x

[jora70072-bib-0022] Murayama, K. , Sakaki, M. , Yan, V. X. , & Smith, G. M. (2014). Type I error inflation in the traditional by‐participant analysis to metamemory accuracy: A generalized mixed‐effects model perspective. Journal of Experimental Psychology: Learning, Memory, and Cognition, 40(5), 1287–1306. 10.1037/a0036914 24911135

[jora70072-bib-0023] Neal, J. W. (2025). How many friends do youth nominate? A meta‐analysis of gender, age, and geographic differences in average outdegree centrality. Social Networks, 80, 65–75. 10.1016/j.socnet.2024.10.001

[jora70072-bib-0024] Nelson, T. O. (1990). Metamemory: A theoretical framework and new findings. In G. H. Bower (Ed.), Psychology of learning and motivation Vol. 26 (pp. 125–173). Academic Press. 10.1016/S0079-7421(08)60053-5

[jora70072-bib-0025] Niebaum, J. C. , Chevalier, N. , Guild, R. M. , & Munakata, Y. (2021). Developing adaptive control: Age‐related differences in task choices and awareness of proactive and reactive control demands. Cognitive, Affective, & Behavioral Neuroscience, 21(3), 561–572. 10.3758/s13415-020-00832-2 PMC10162508 33009653

[jora70072-bib-0026] Ohtani, K. , & Hisasaka, T. (2018). Beyond intelligence: A meta‐analytic review of the relationship among metacognition, intelligence, and academic performance. Metacognition and Learning, 13(2), 179–212. 10.1007/s11409-018-9183-8

[jora70072-bib-0027] Okita, S. Y. (2014). Learning from the folly of others: Learning to self‐correct by monitoring the reasoning of virtual characters in a computer‐supported mathematics learning environment. Computers & Education, 71, 257–278. 10.1016/j.compedu.2013.09.018

[jora70072-bib-0028] Paulus, M. , Tsalas, N. , Proust, J. , & Sodian, B. (2014). Metacognitive monitoring of oneself and others: Developmental changes during childhood and adolescence. Journal of Experimental Child Psychology, 122, 153–165. 10.1016/j.jecp.2013.12.011 24607803

[jora70072-bib-0029] Rambaran, J. A. , Hopmeyer, A. , Schwartz, D. , Steglich, C. , Badaly, D. , & Veenstra, R. (2017). Academic functioning and peer influences: A short‐term longitudinal study of network–behavior dynamics in middle adolescence. Child Development, 88(2), 523–543. 10.1111/cdev.12611 27580016

[jora70072-bib-0030] Ripley, R. M. , Snijders, T. A. B. , Boda, Z. , Vörös, A. , & Preciado, P. (2024). Manual for RSiena . https://www.stats.ox.ac.uk/~snijders/siena/RSiena_Manual.pdf

[jora70072-bib-0031] Roebers, C. M. (2017). Executive function and metacognition: Towards a unifying framework of cognitive self‐regulation. Developmental Review, 45, 31–51. 10.1016/j.dr.2017.04.001

[jora70072-bib-0050] Roebers, C. M. , & Spiess, M. (2017). The development of metacognitive monitoring and control in second graders: A short‐term longitudinal study. Journal of Cognition and Development, 18(1), 110–128. 10.1080/15248372.2016.1157079

[jora70072-bib-0032] Roebers, C. M. , Mayer, B. , Steiner, M. , Bayard, N. S. , & van Loon, M. H. (2019). The role of children's metacognitive experiences for cue utilization and monitoring accuracy: A longitudinal study. Developmental Psychology, 55(10), 2077–2089. 10.1037/dev0000776 31343230

[jora70072-bib-0033] Rosseel, Y. (2012). Lavaan: An R package for structural equation modeling. Journal of Statistical Software, 48(2), 1–36. 10.18637/jss.v048.i02

[jora70072-bib-0034] Schneider, W. , & Löffler, E. (2016). The development of metacognitive knowledge in children and adolescents. In J. Dunlosky & R. A. Bjork (Eds.), The Oxford handbook of Metamemory (pp. 191–517). Oxford University Press.

[jora70072-bib-0035] Schwartz, D. , Kelly, B. M. , & Duong, M. T. (2013). Do academically‐engaged adolescents experience social sanctions from the peer group? Journal of Youth and Adolescence, 42, 1319–1330. 10.1007/s10964-012-9882-4 23277293

[jora70072-bib-0036] Shin, H. (2018). The role of friends in help‐seeking tendencies during early adolescence: Do classroom goal structures moderate selection and influence of friends? Contemporary Educational Psychology, 53, 135–145. 10.1016/j.cedpsych.2018.03.002

[jora70072-bib-0037] Shin, H. , & Ryan, A. M. (2014). Early adolescent friendships and academic adjustment: Examining selection and influence processes with longitudinal social network analysis. Developmental Psychology, 50(11), 2462–2472. 10.1037/a0037922 25221841

[jora70072-bib-0038] Snijders, T. A. B. , Van De Bunt, G. G. , & Steglich, C. E. G. (2010). Introduction to stochastic actor‐based models for network dynamics. Social Networks, 32(1), 44–60. 10.1016/j.socnet.2009.02.004

[jora70072-bib-0039] Stadtfeld, C. , Snijders, T. A. B. , Steglich, C. , & Van Duijn, M. (2020). Statistical power in longitudinal network studies. Sociological Methods & Research, 49(4), 1103–1132. 10.1177/0049124118769113

[jora70072-bib-0040] van Ginkel, J. R. , Linting, M. , Rippe, R. C. A. , & van der Voort, A. (2020). Rebutting existing misconceptions about multiple imputation as a method for handling missing data. Journal of Personality Assessment, 102(3), 297–308. 10.1080/00223891.2018.1530680 30657714

[jora70072-bib-0041] van Loon, M. , Orth, U. , & Roebers, C. (2024). The structure of metacognition in middle childhood: Evidence for a unitary metacognition‐for‐memory factor. Journal of Experimental Child Psychology, 241, 105857. 10.1016/j.jecp.2023.105857 38241971

[jora70072-bib-0043] van Loon, M. H. , Bayard, N. S. , Steiner, M. , & Roebers, C. M. (2021). Connecting teachers' classroom instructions with children's metacognition and learning in elementary school. Metacognition and Learning, 16(3), 623–650. 10.1007/s11409-020-09248-2 34867118 PMC8616875

[jora70072-bib-0044] van Loon, M. H. , Bayard, N. S. , Steiner, M. , & Roebers, C. M. (2022). The accuracy and annual rank‐order stability of elementary school children's self‐monitoring judgments. Journal of Applied Developmental Psychology, 80, 101419. 10.1016/j.appdev.2022.101419

[jora70072-bib-0045] van Loon, M. H. , & Oeri, N. S. (2023). Examining on‐task regulation in school children: Interrelations between monitoring, regulation, and task performance. Journal of Educational Psychology, 115(3), 446–459. 10.1037/edu0000781

[jora70072-bib-0046] van Loon, M. H. , & Roebers, C. M. (2017). Effects of feedback on self‐evaluations and self‐regulation in elementary school. Applied Cognitive Psychology, 31(5), 508–519. 10.1002/acp.3347

[jora70072-bib-0047] Vosylis, R. , Erentaitė, R. , Simonaitienė, B. , Melnikė, E. , Sevalneva, D. , Morkevičius, V. , Žvaliauskas, G. , & Hemker, B. (2024). Unpacking grading bias in middle school: A multilevel analysis of grade vs. test discrepancy considering student and school characteristics. Studies in Educational Evaluation, 83, 101398. 10.1016/j.stueduc.2024.101398

[jora70072-bib-0048] Wang, M.‐T. , Kiuru, N. , Degol, J. L. , & Salmela‐Aro, K. (2018). Friends, academic achievement, and school engagement during adolescence: A social network approach to peer influence and selection effects. Learning and Instruction, 58, 148–160. 10.1016/j.learninstruc.2018.06.003

[jora70072-bib-0049] Zimmerman, B. J. (2002). Becoming a self‐regulated learner: An overview. Theory Into Practice, 41(2), 64–70. 10.1207/s15430421tip4102_2

