# Supplementary material for: A short‐term longitudinal study linking adolescents' metacognition, learning, and social friendship networks
Source: J Res Adolesc. 2025 Aug 28;35(3):e70072. doi: 10.1111/jora.70072 (PMC12392705; doi:10.1111/jora.70072)

# Online Supplementary Materials

Contents

[Distributions of the attribute data and categorization (cut-off values) 1](#_Toc188627753)

[Information about the categorization of the attribute data 1](#_Toc188627754)

[Monitoring Accuracy 3](#_Toc188627755)

[Monitoring-Based-Restudy 4](#_Toc188627756)

[Decision Accuracy 6](#_Toc188627757)

[Task Scores 7](#_Toc188627758)

[Graphs indicating goodness of fit for the longitudinal social network analyses 8](#_Toc188627759)

[Monitoring Accuracy 8](#_Toc188627760)

[Monitoring-Based-Restudy 11](#_Toc188627761)

[Decision Accuracy 13](#_Toc188627763)

[Task Scores 16](#_Toc188627764)

# Distributions of the attribute data and binning (cut-off values)

## Information about the categorization of the attribute data

For the task score variable, 32.5% of participants achieved the highest possible score at T1, and 35.3% at T2. Given these distributions, three bins appeared optimal, ensuring that the groups had similar sample sizes. We therefore categorized the data for task scores, monitoring accuracy, and monitoring-based restudy in three bins, reflecting low, medium, and high scores on the specific attributes. However, for decision accuracy, 50% of participants had the highest decision accuracy scores at T1, and 43.6% at T2. Due to this portion of participants scoring at the highest level, we only binned the decision accuracy data into two categories (low and high accuracy), to create bins of approximately equal sizes.

The binning was based on the data distribution at the second time point (T2). For the binned attribute variables, we then applied the same cutoff values for T1 as for T2, ensuring that individual changes between categories over time reflected actual changes in underlying values.

Further information about the distribution and categorization of each of the attribute variables is provided below.

## Monitoring Accuracy

Distributions:
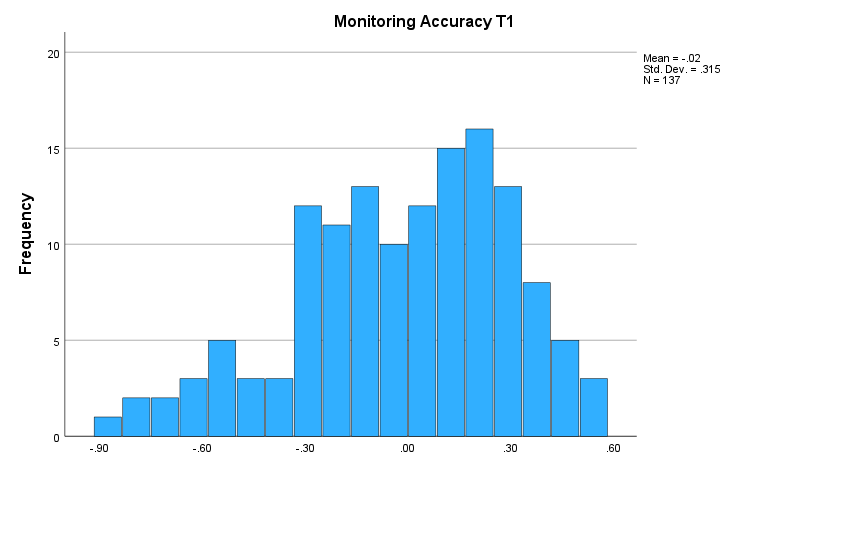

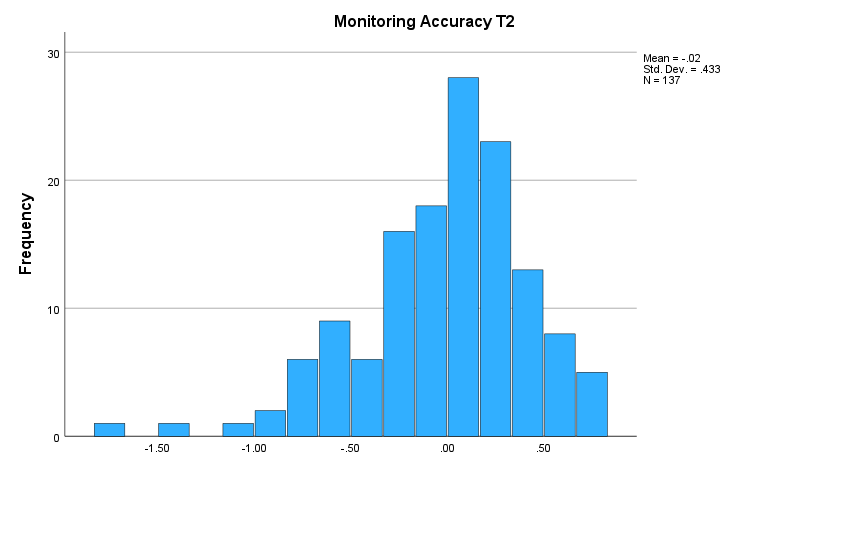


Monitoring Accuracy Categorization:

< -0.15 = 1 (low)
-0.15 – 0.19 = 2 (medium)
> 0.19 = 3 (high)

## Monitoring-Based-Restudy

Distributions:


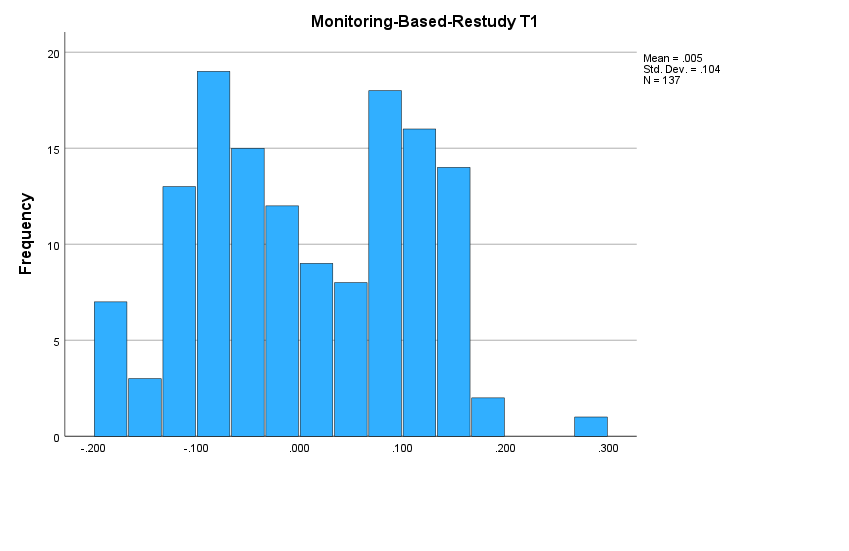


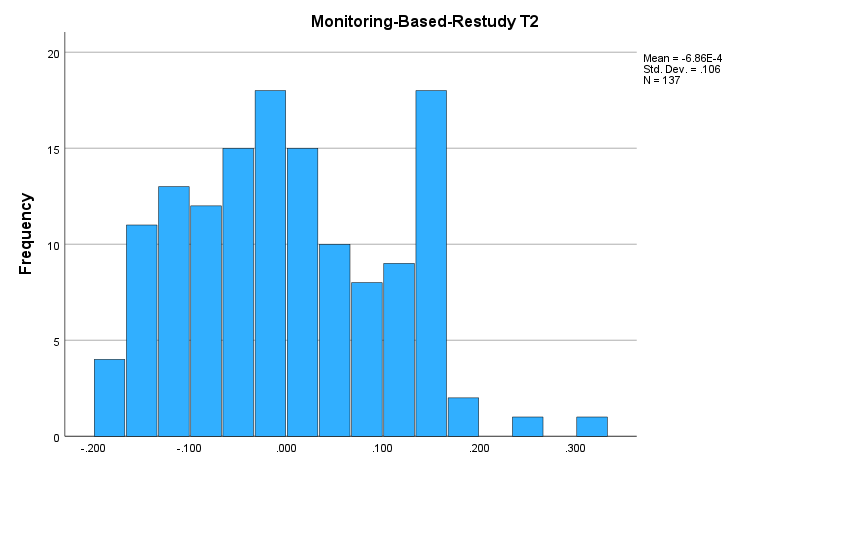


Monitoring-Based-Restudy Categorization (note that higher values indicate less effective monitoring-based-restudy):

> 0.04 = 1 (low)
-0.054 – 0.04 = 2 (medium)
< -0.054 = 1 (high)

## Decision Accuracy

Distributions:

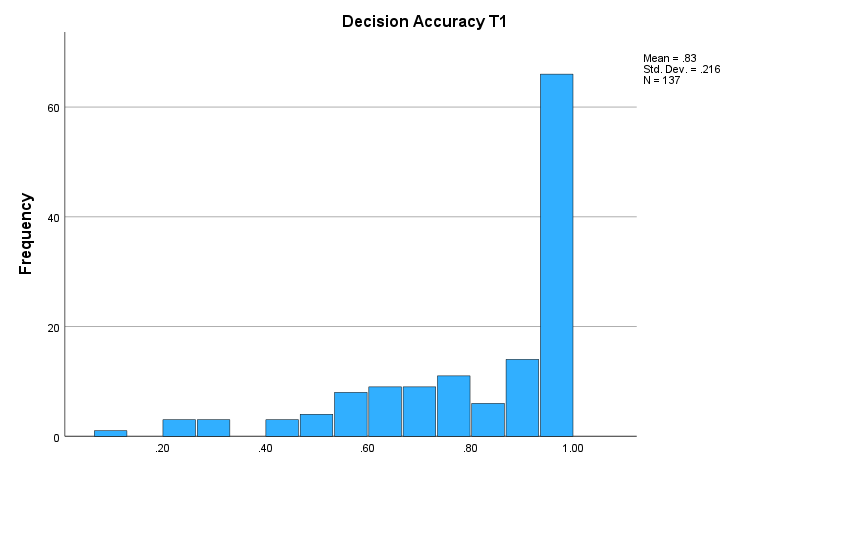


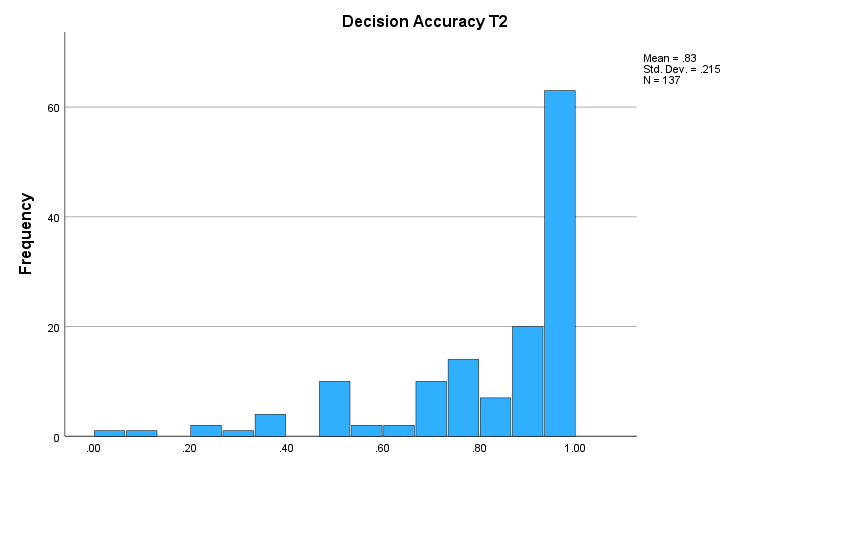


Decision Accuracy Categorization:

< .91 = low
> .91 = high

## Task Scores


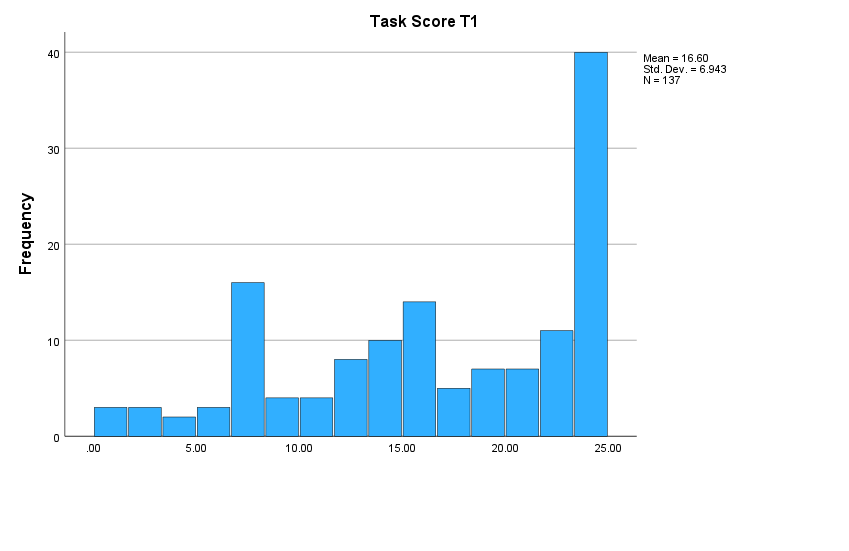

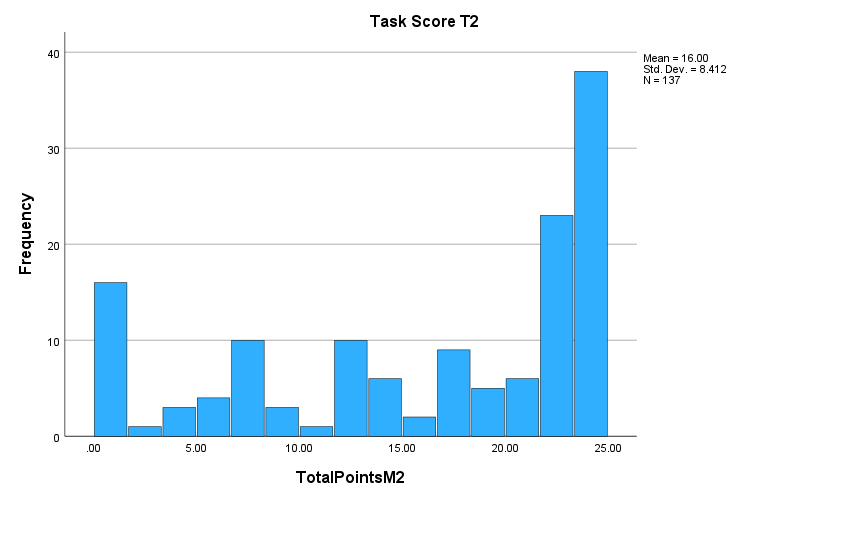


Task Score Categorization:

< 12.31 = 1 (low)
12.31 – 22.08 = 2 (medium)
> 22.08 = 3 (high)

# Graphs indicating goodness of fit for the longitudinal social network analyses

## Monitoring Accuracy


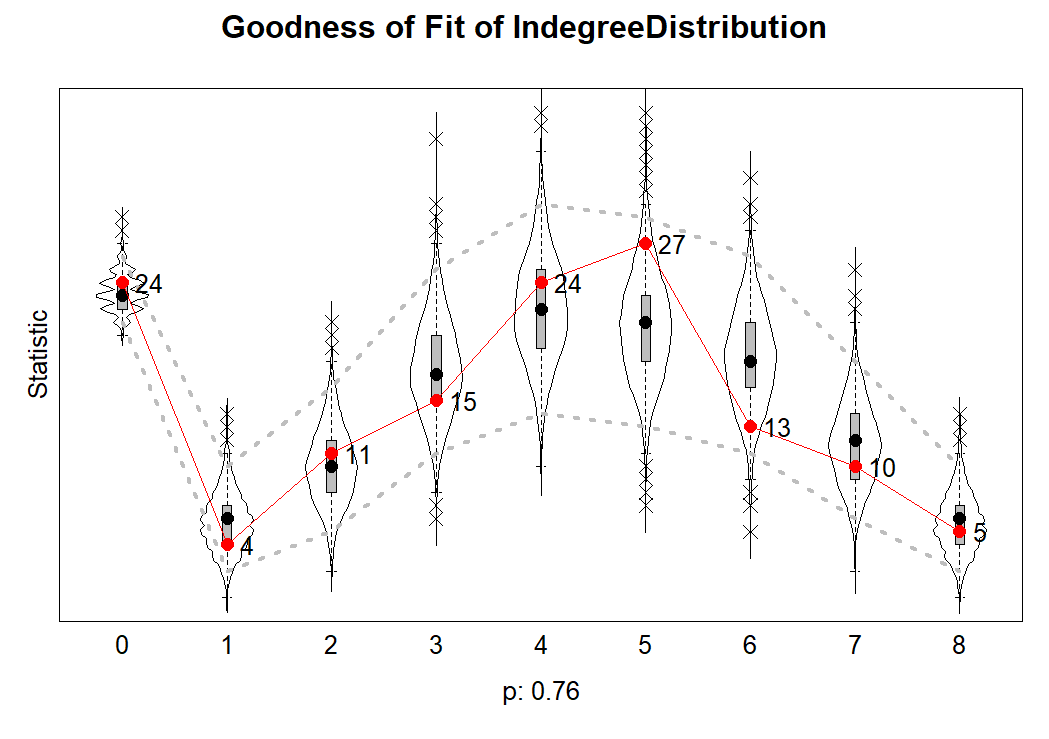


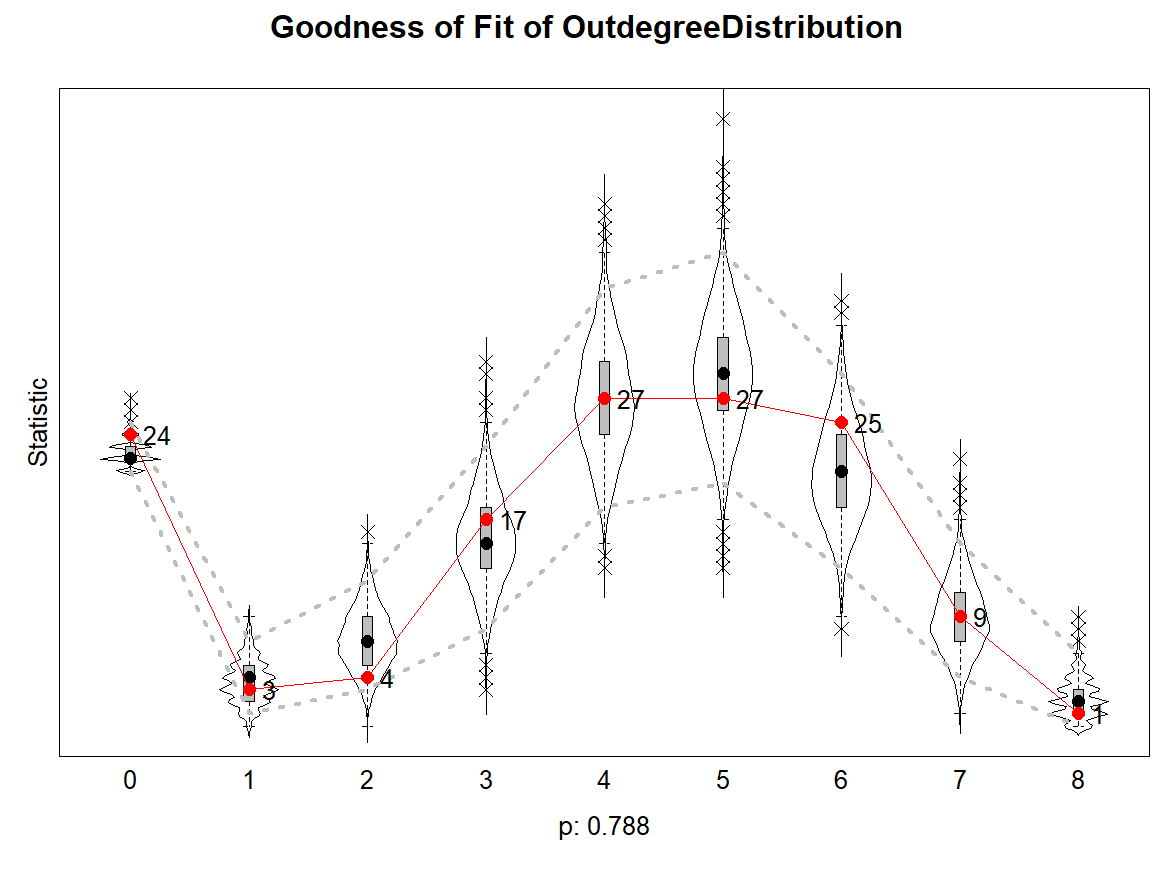


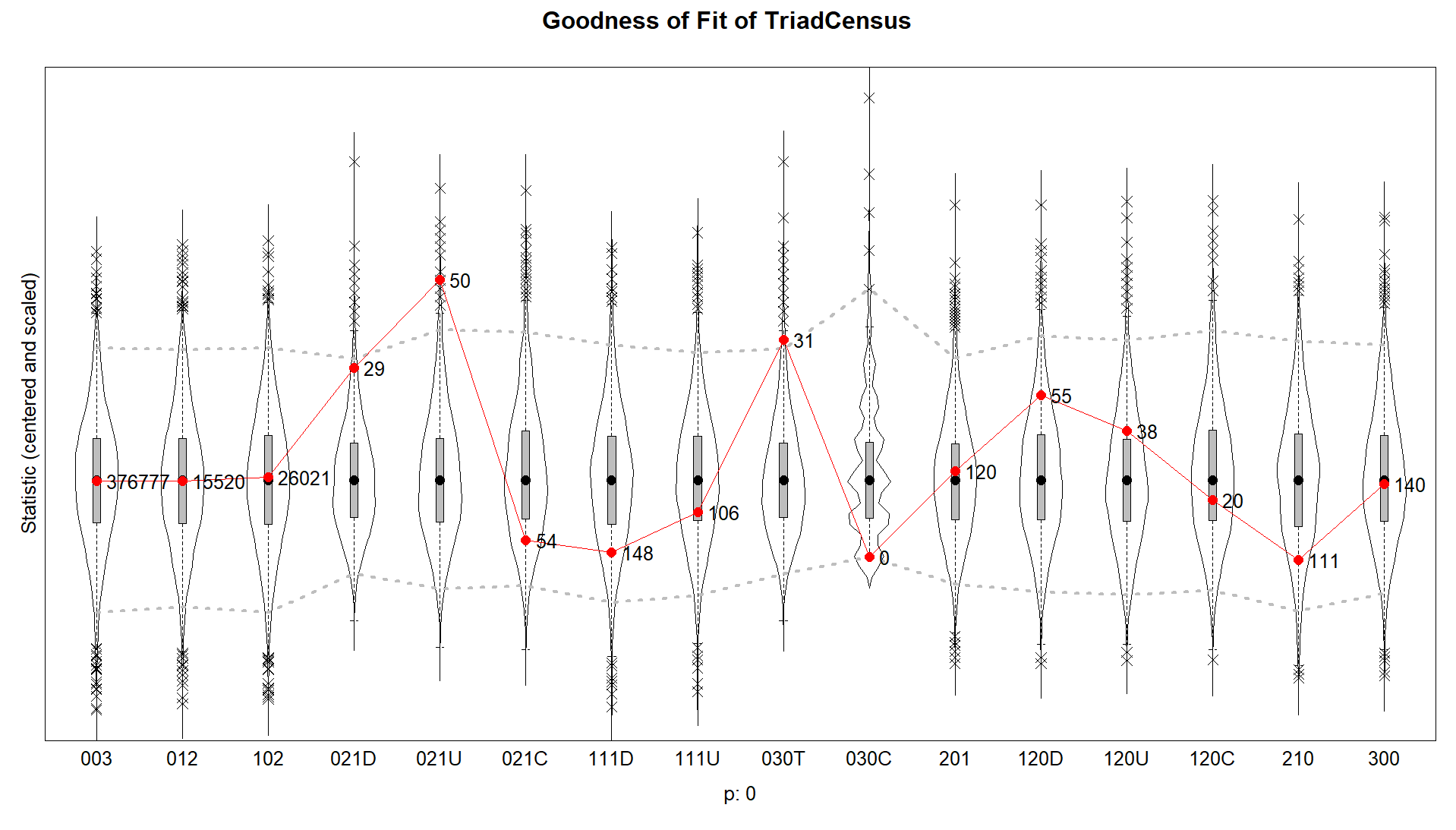


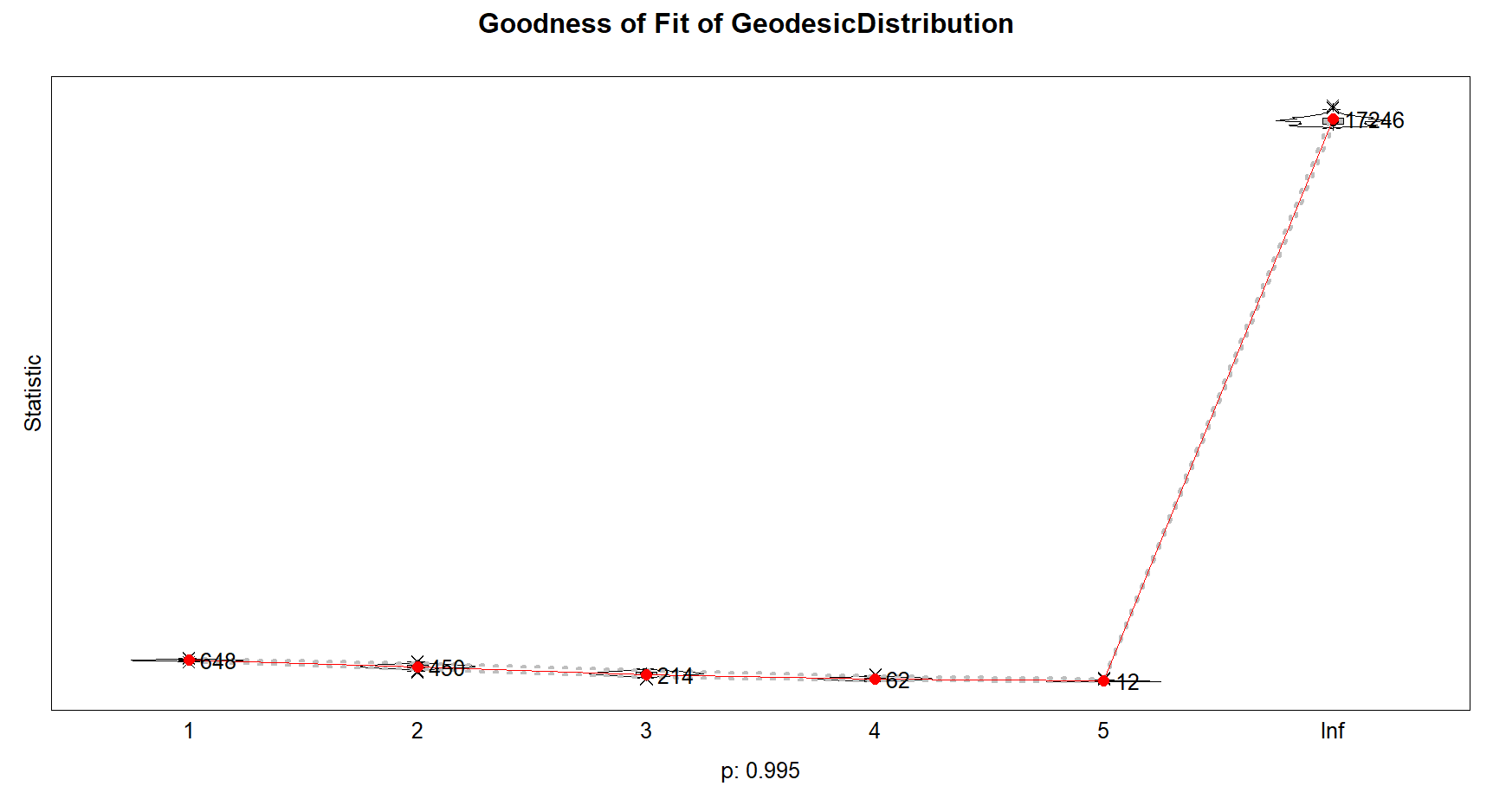


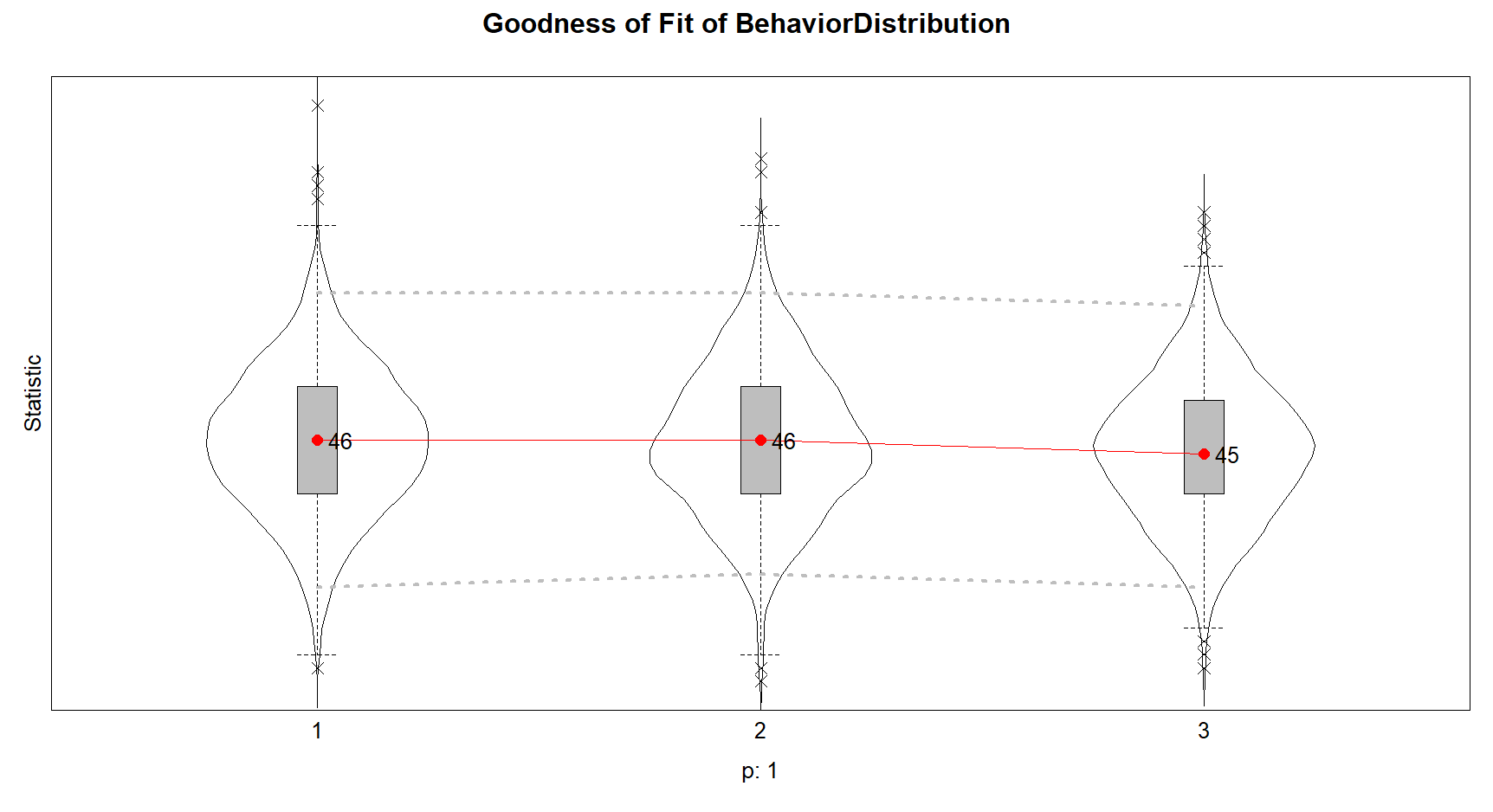


## Monitoring-Based-Restudy


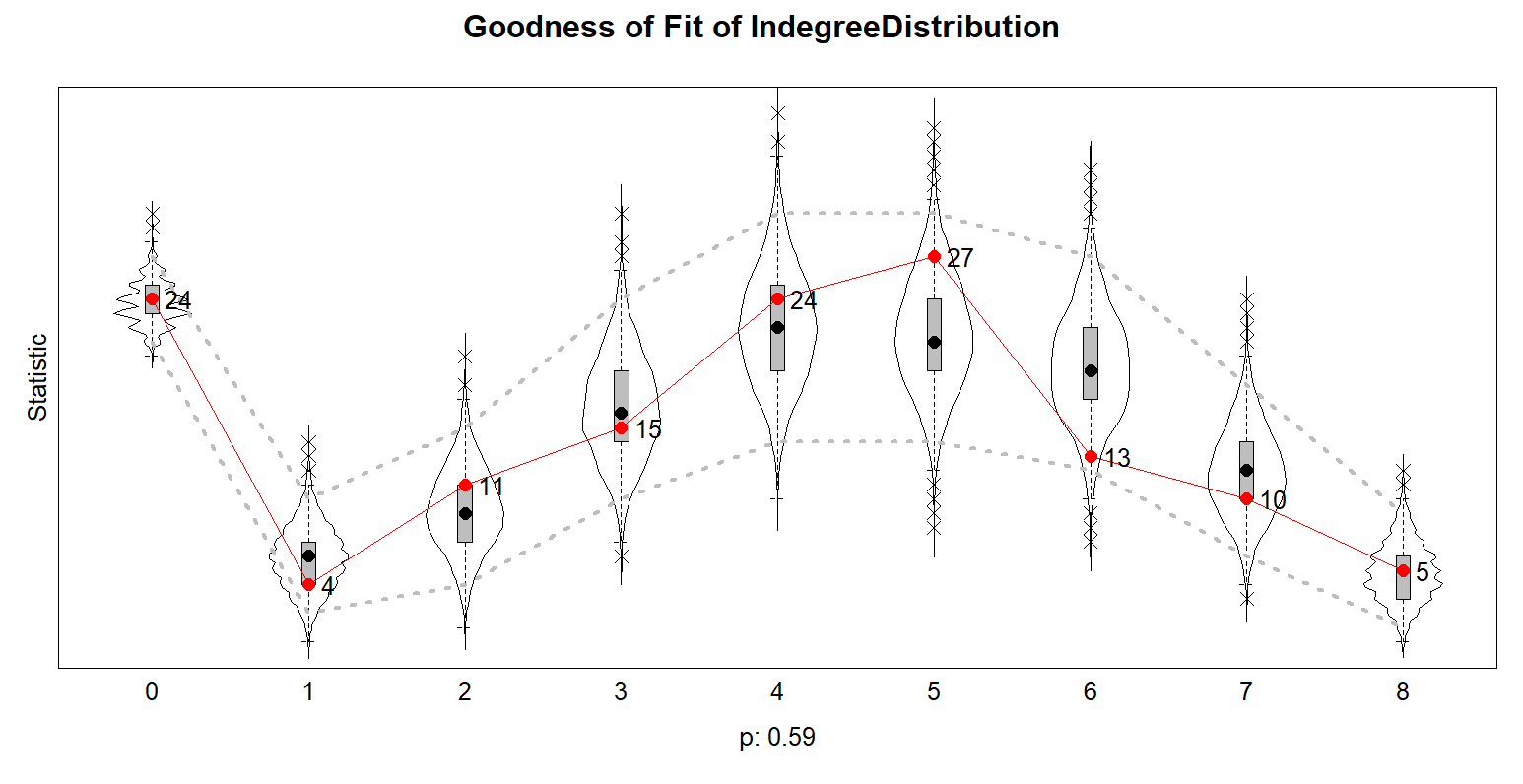


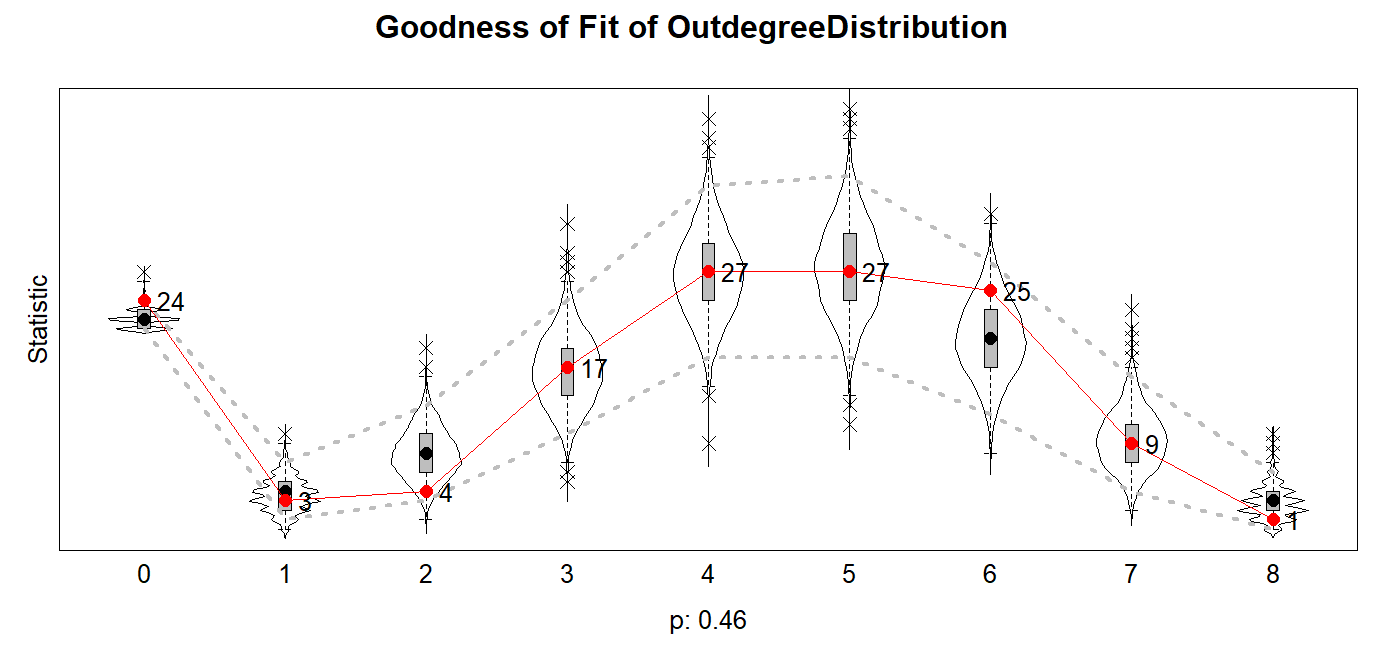


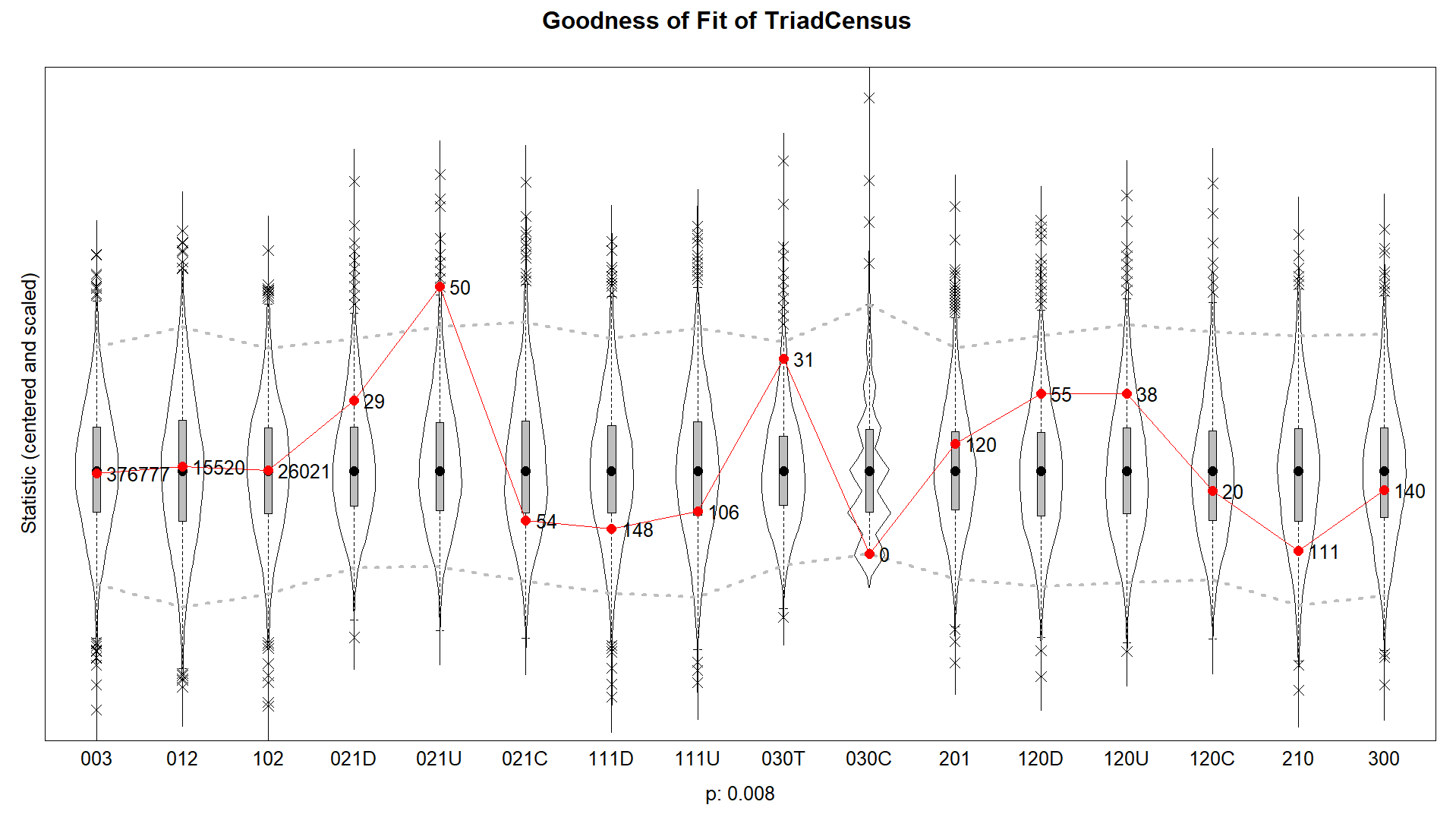


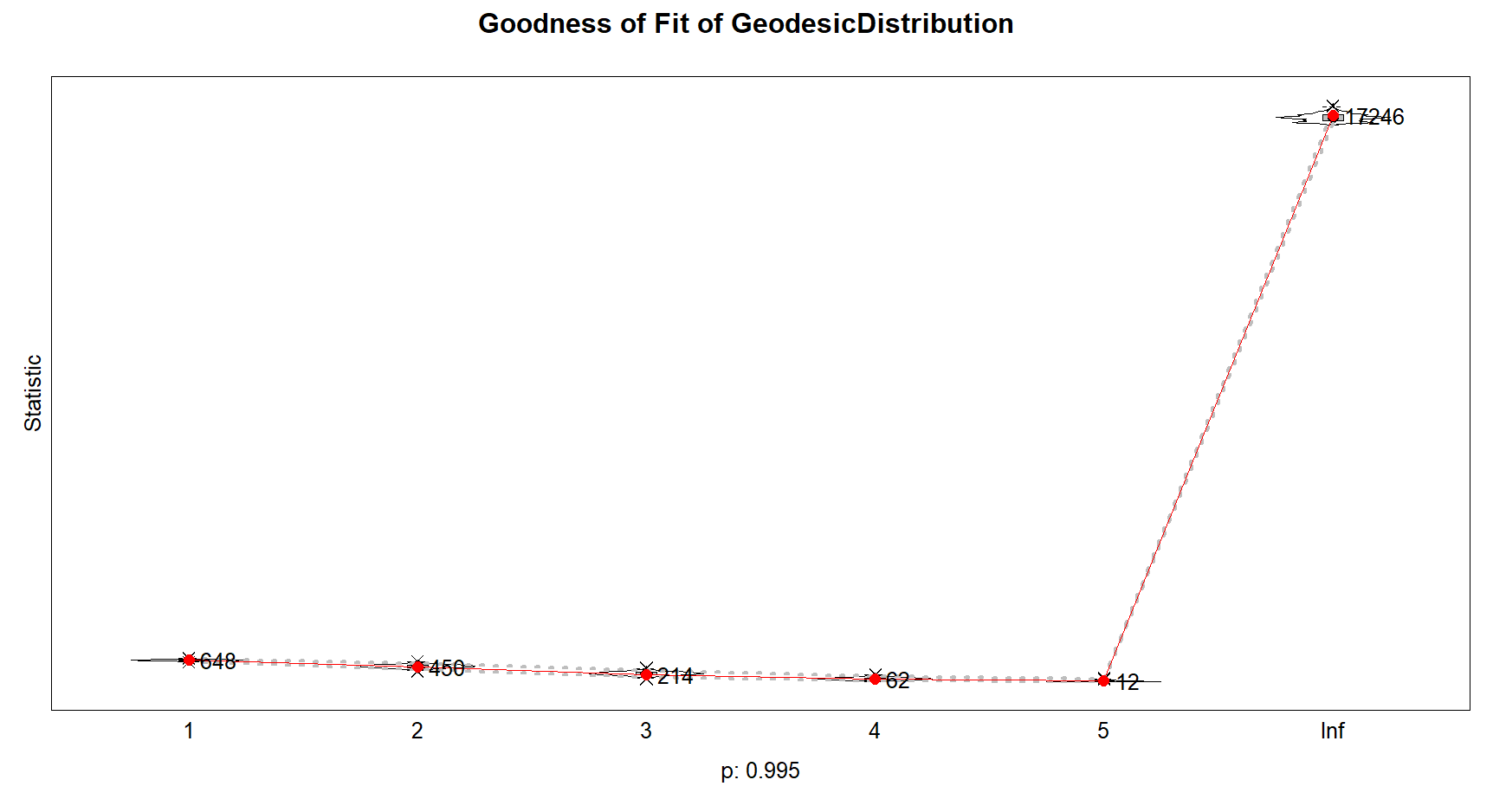


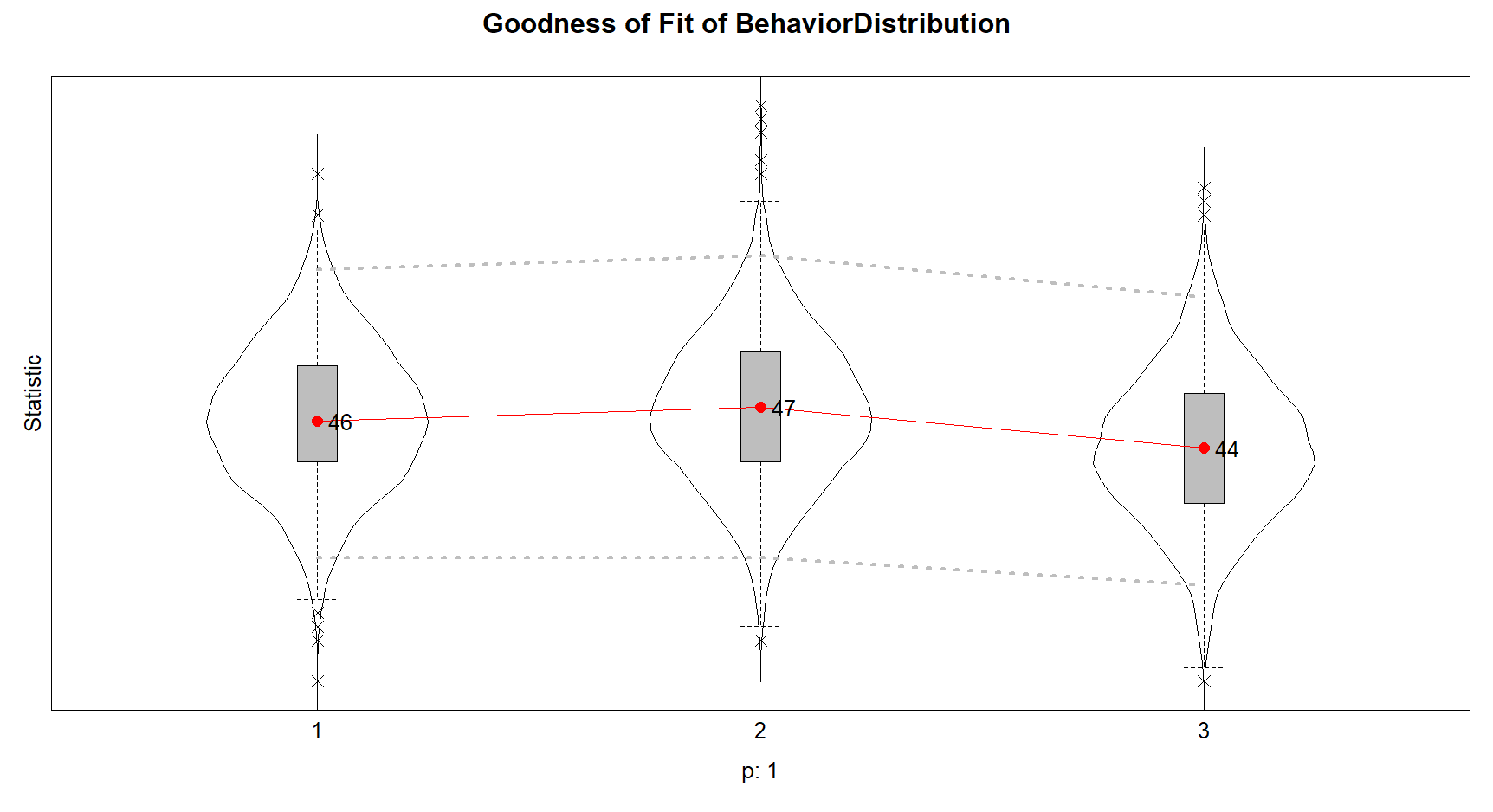


## Decision Accuracy


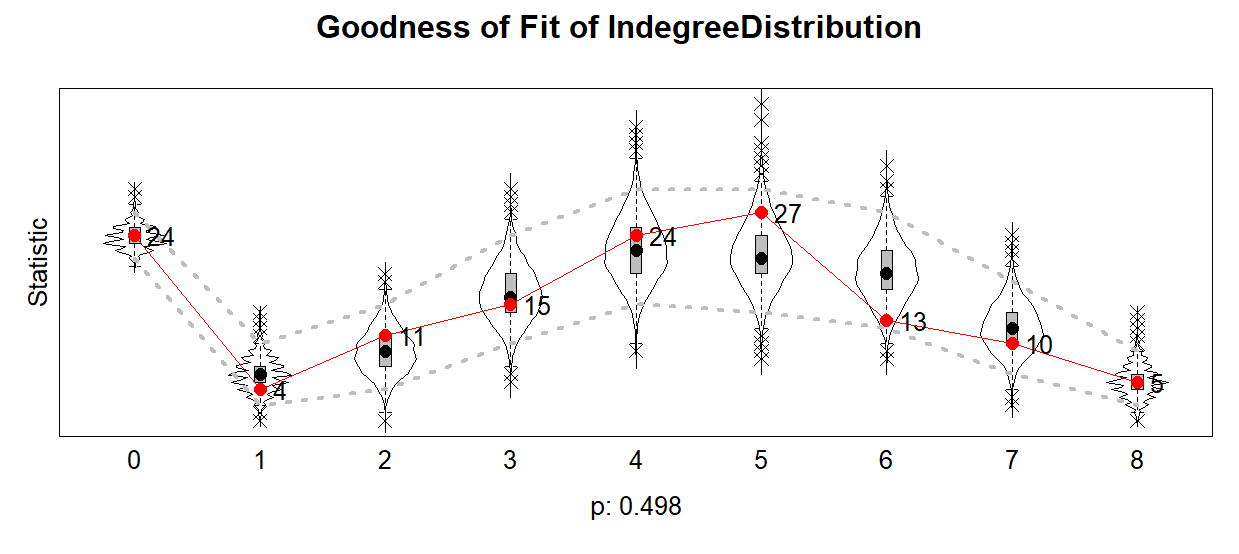


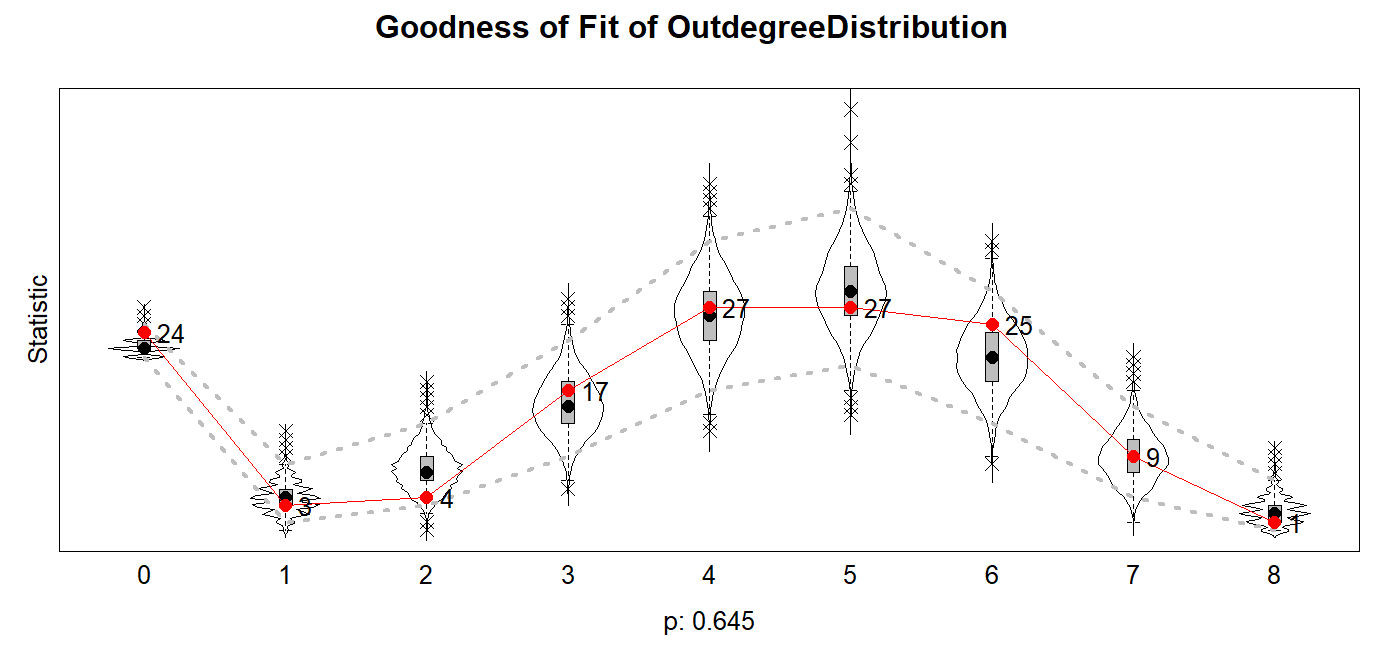


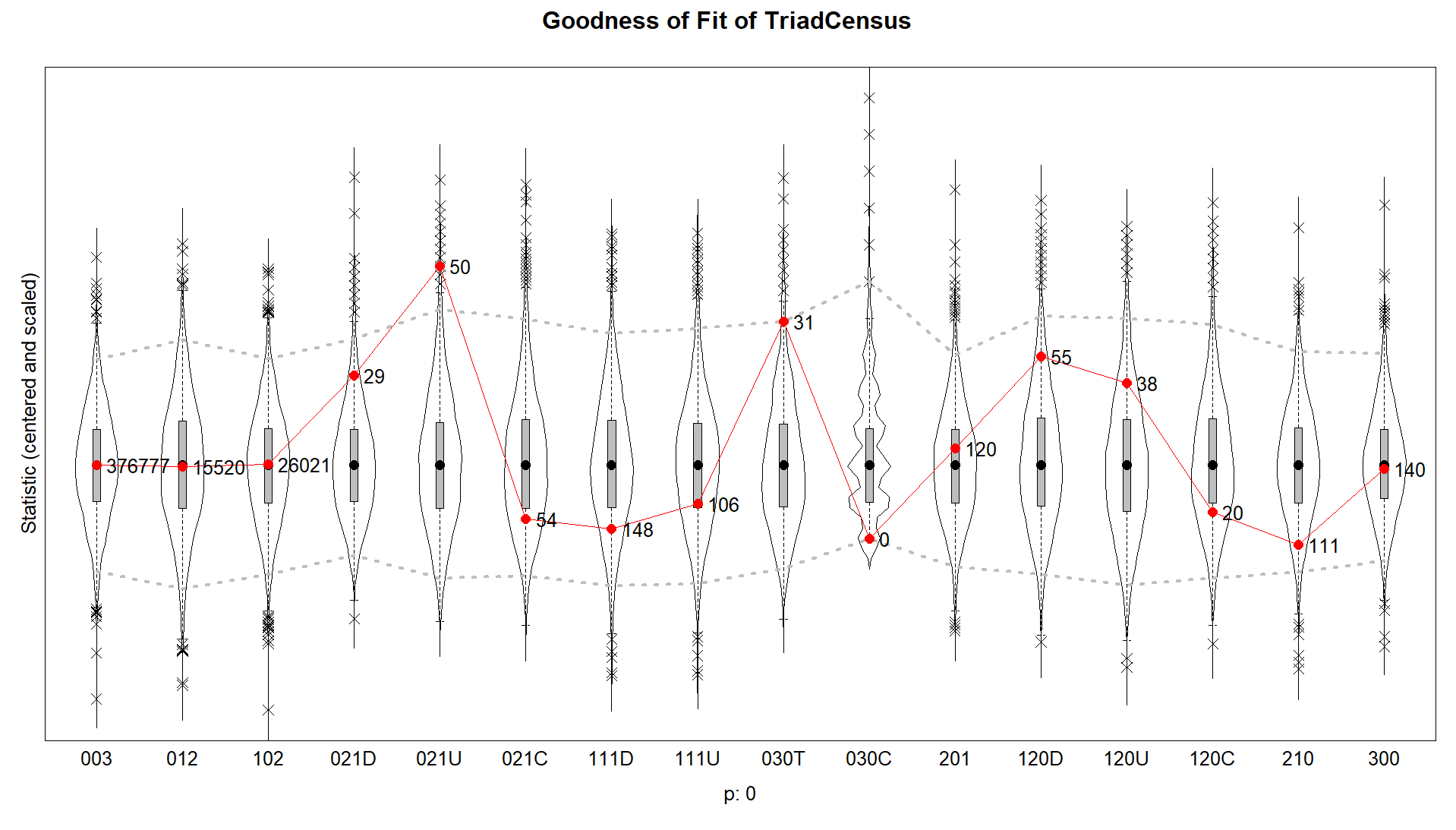


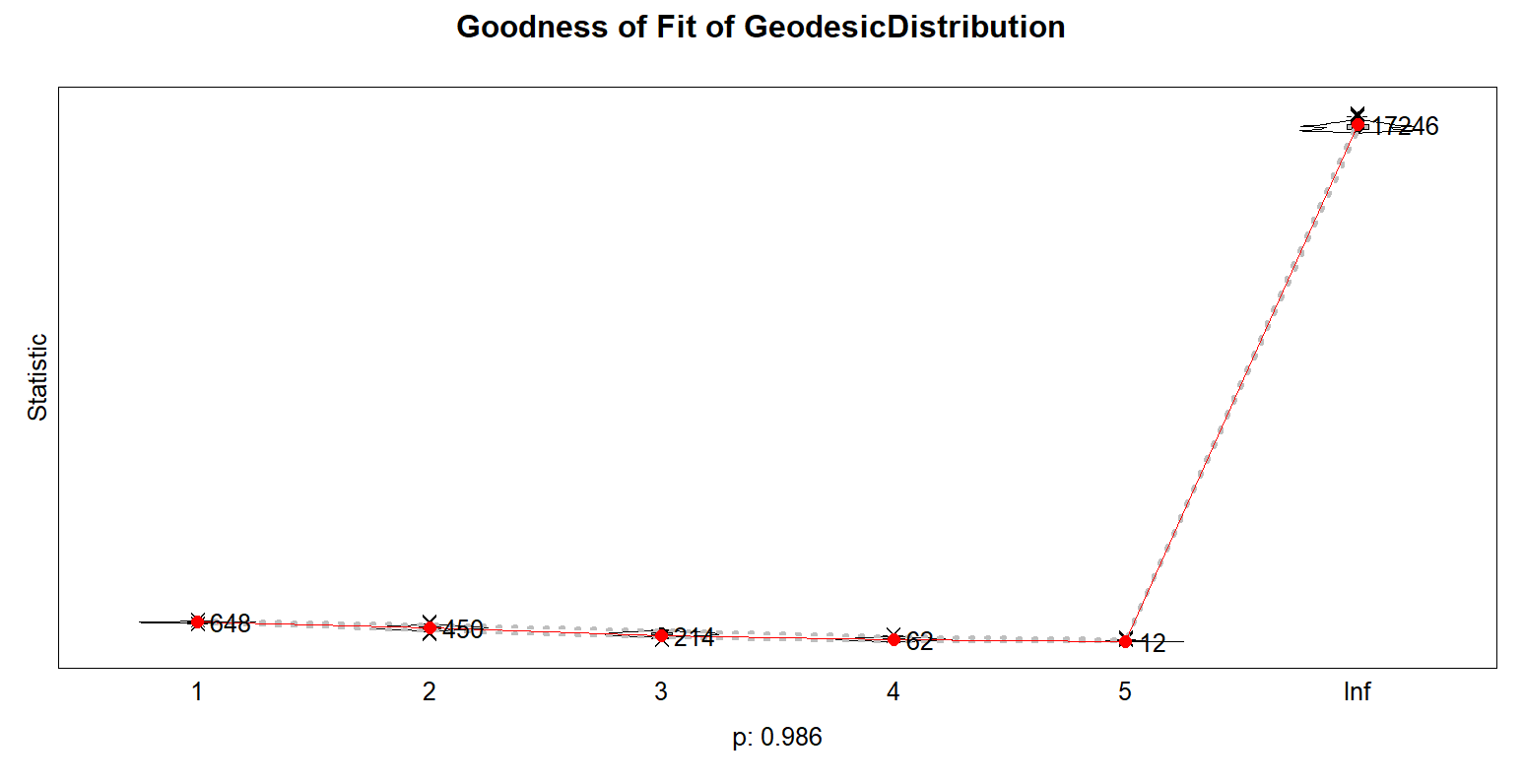


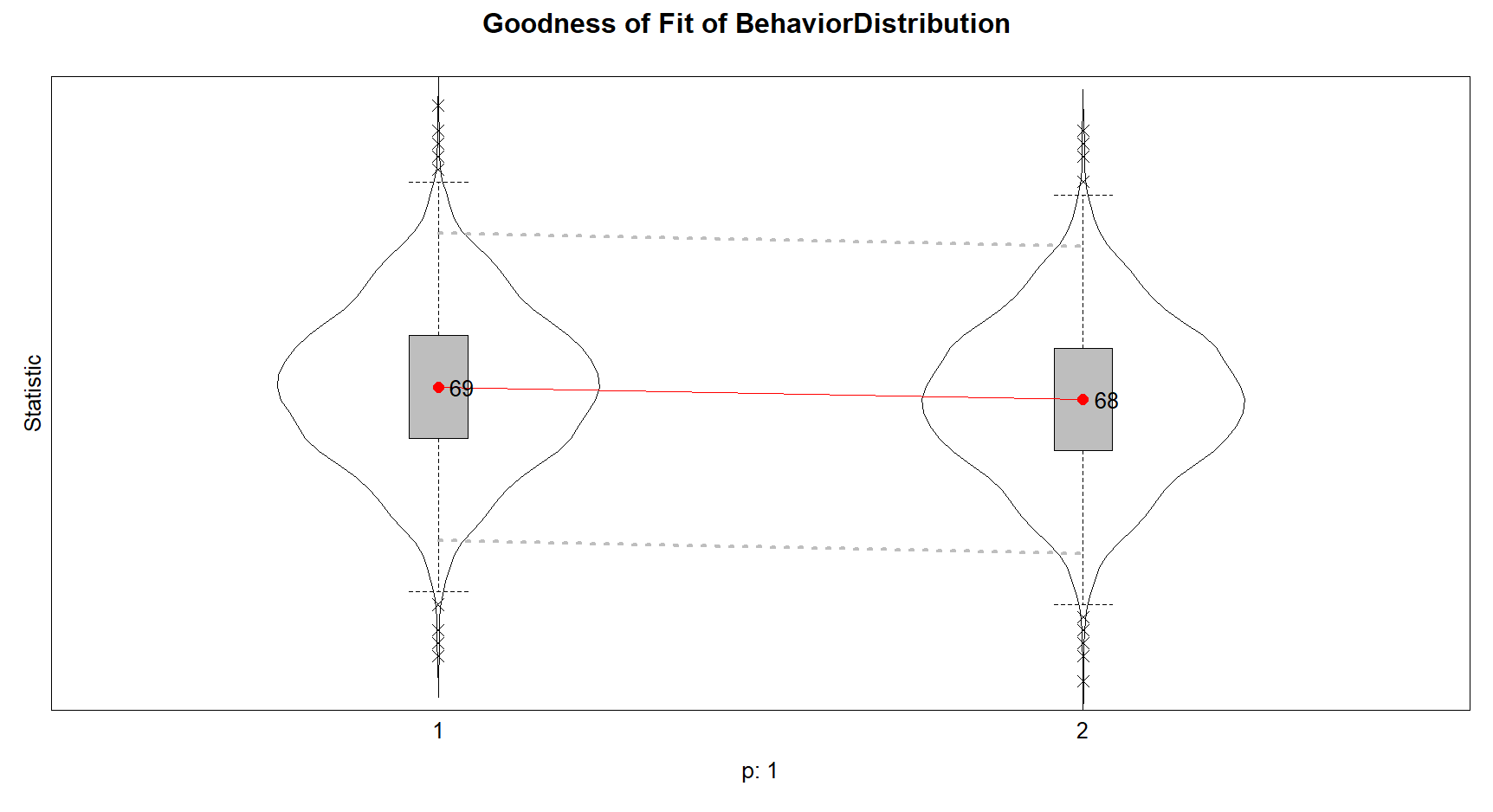


## Task Scores


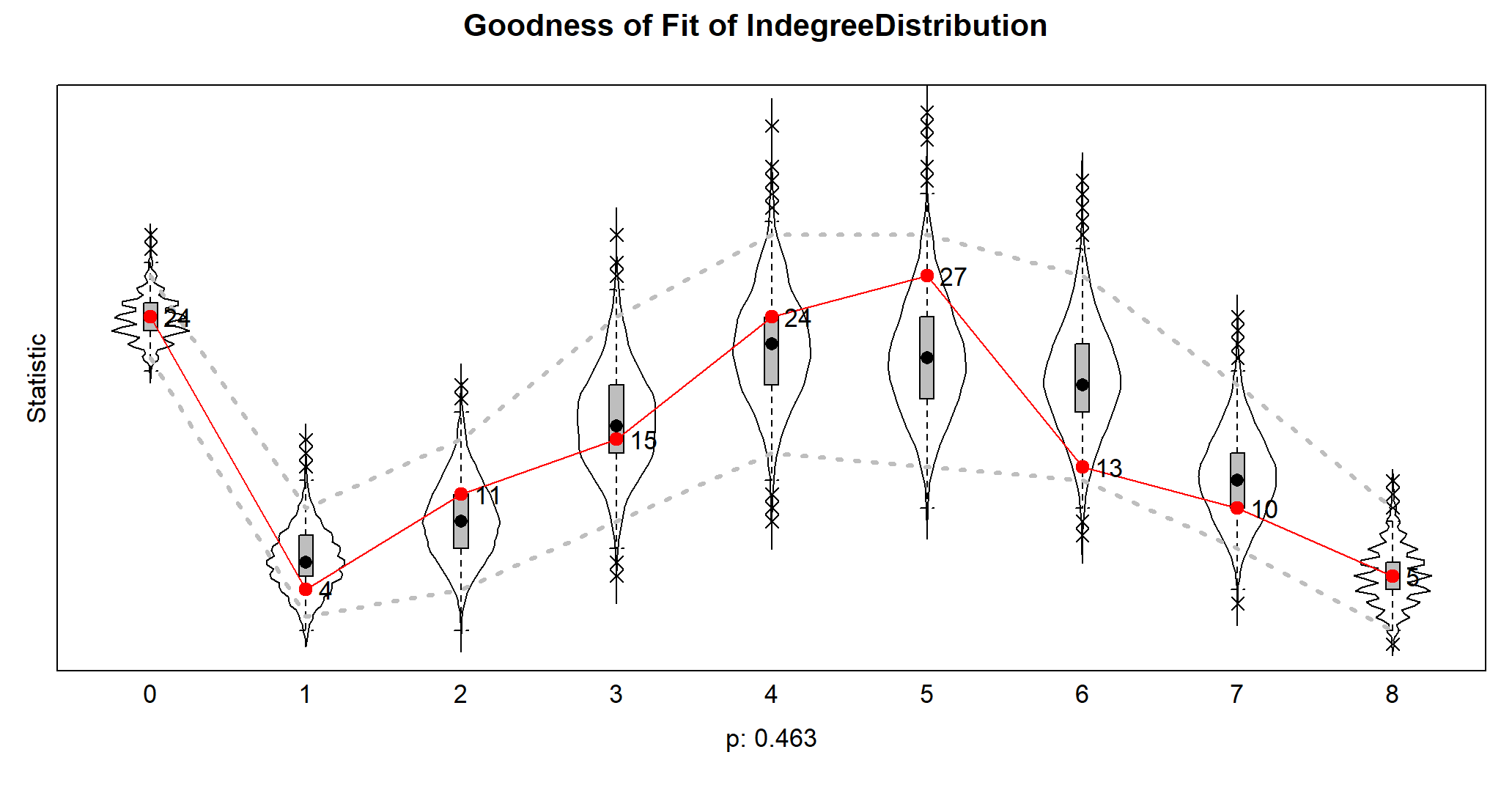


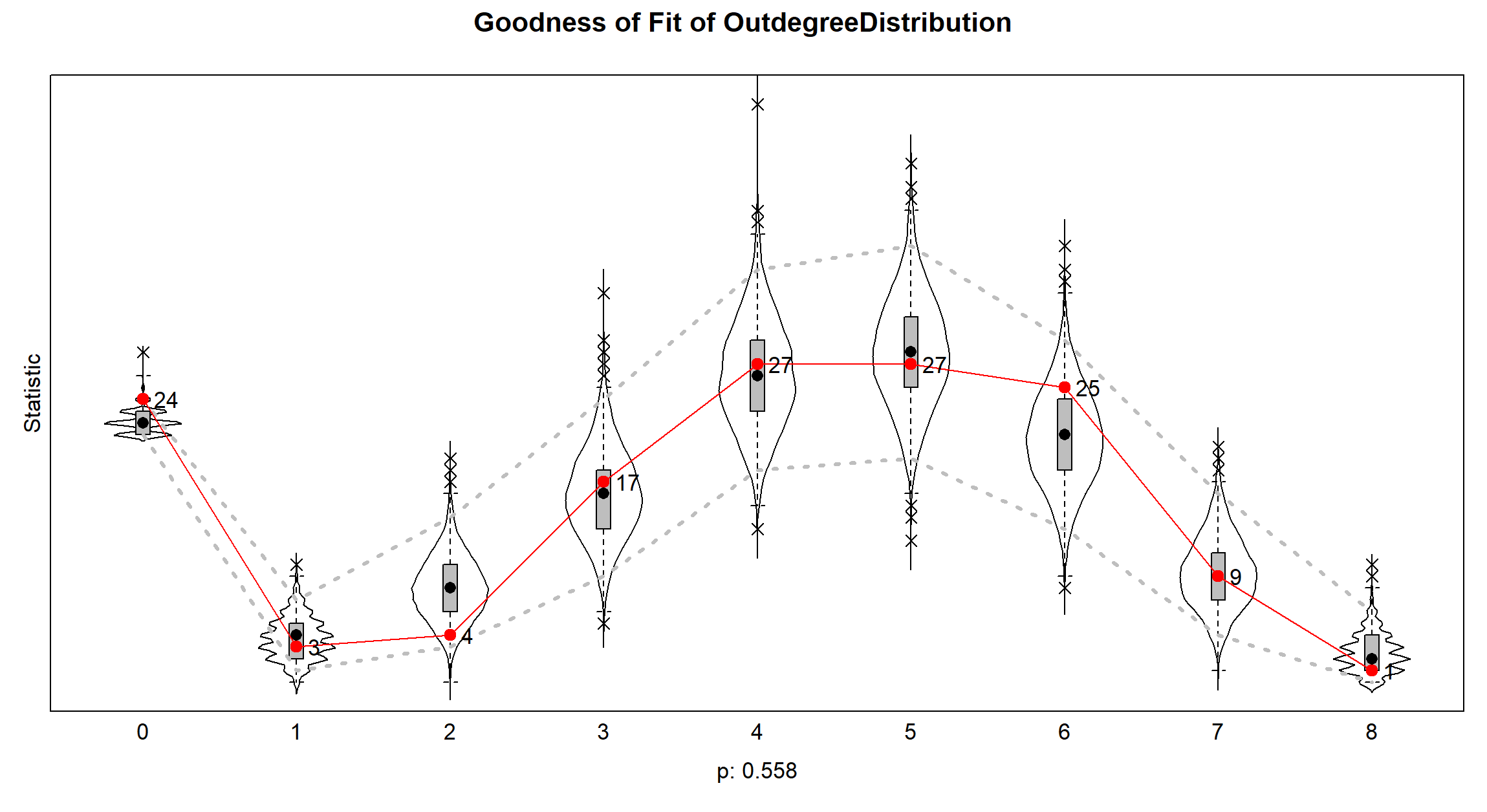


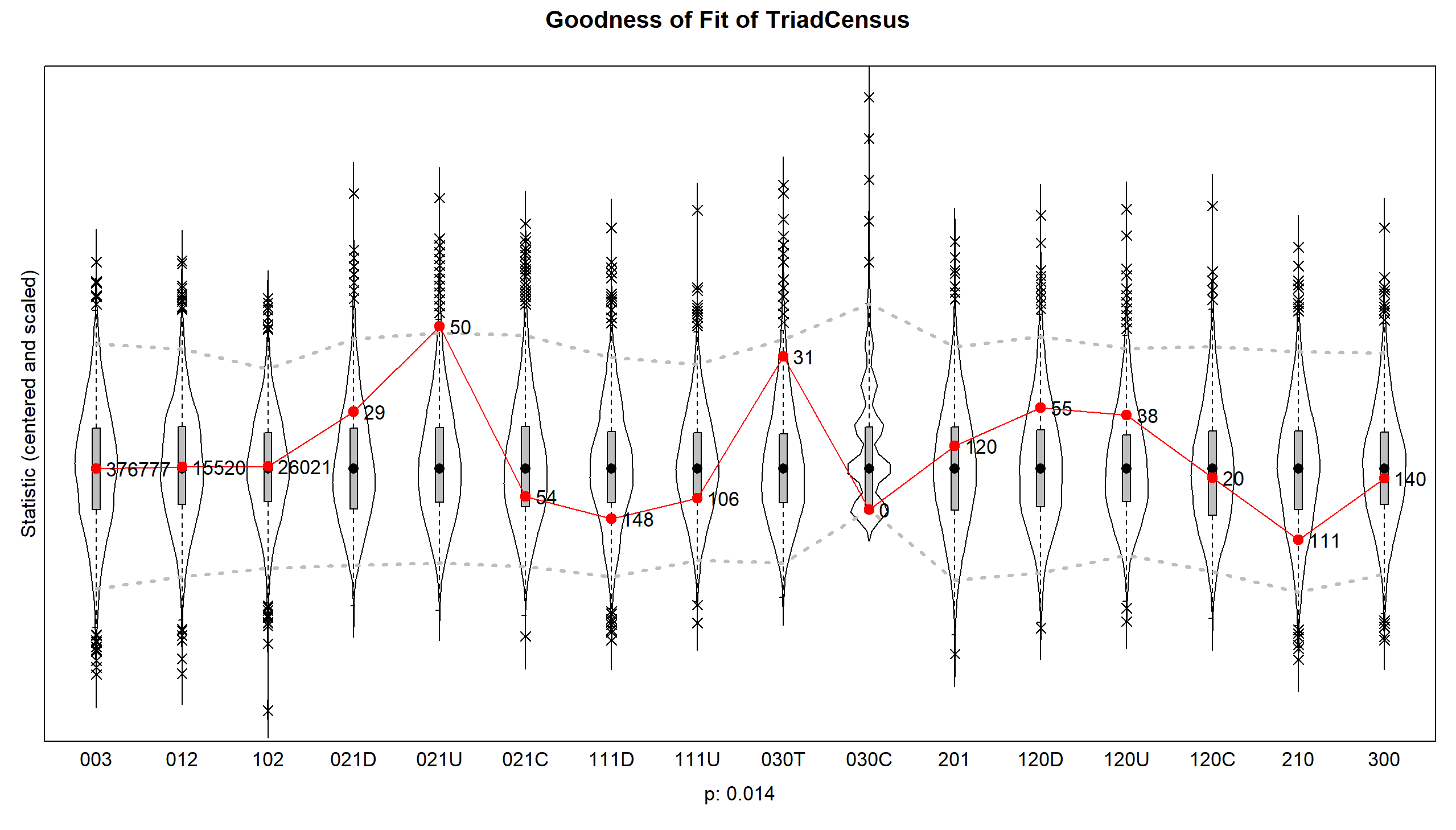


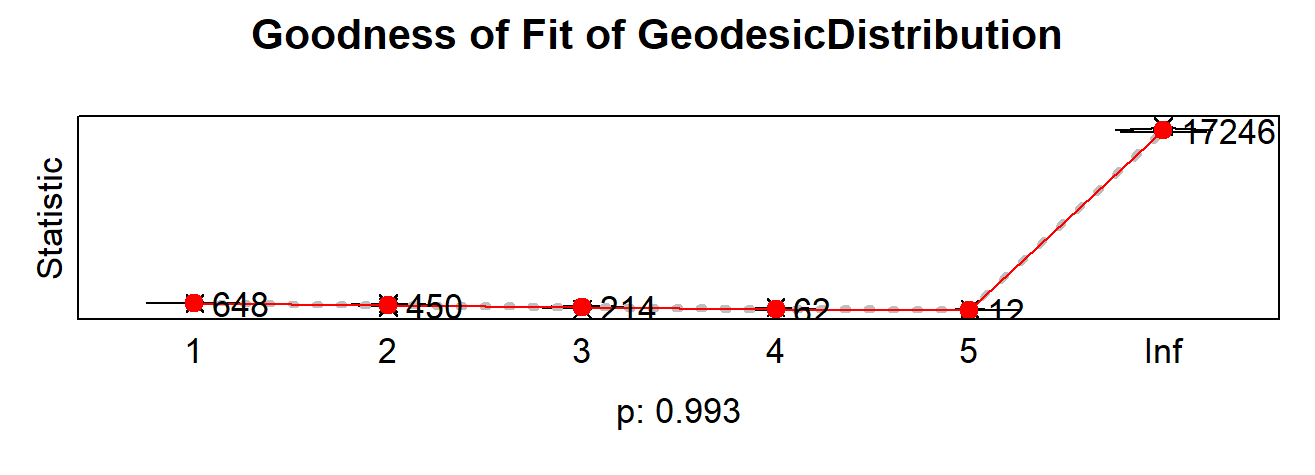


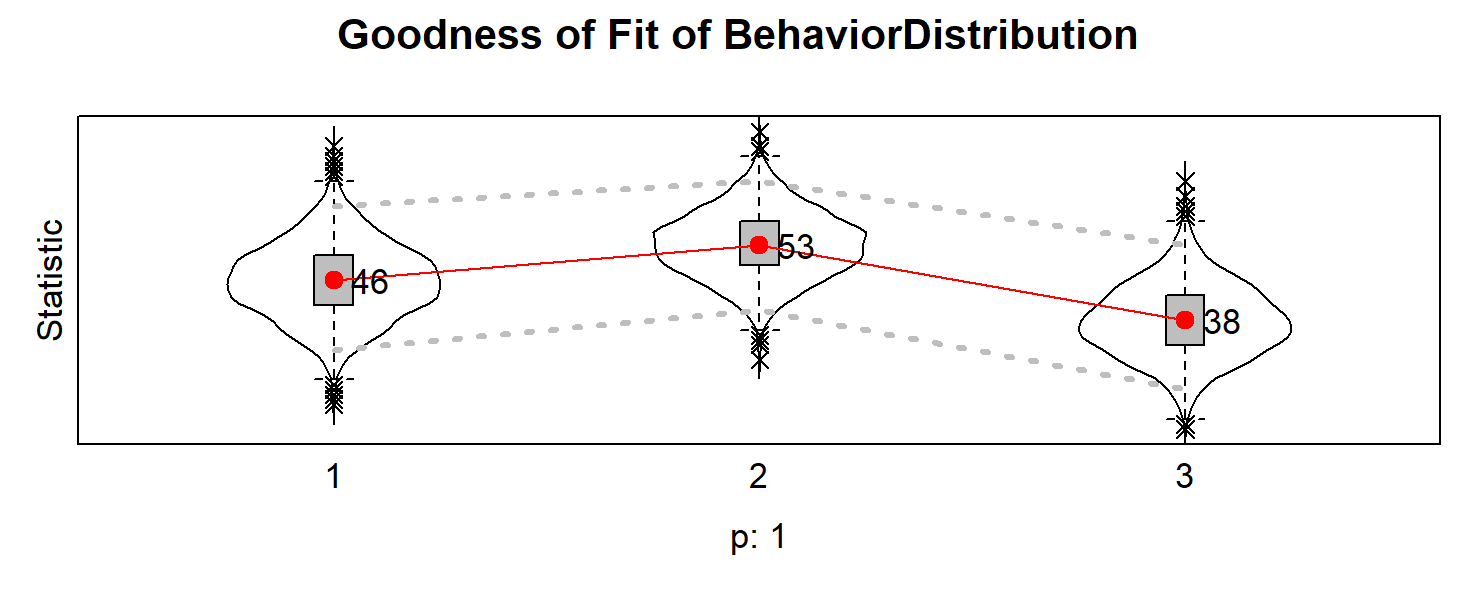

Supplement: Supplementary file 1 — Data S1 [file JORA-35-0-s001.docx]
